# Unveiling Undercover Cropland Inside Forests Using Landscape Variables: A Supplement to Remote Sensing Image Classification

**DOI:** 10.1371/journal.pone.0130079

**Published:** 2015-06-22

**Authors:** Yohannes Ayanu, Christopher Conrad, Anke Jentsch, Thomas Koellner

**Affiliations:** 1 University of Bayreuth, Faculty of Biology, Chemistry and Earth Sciences, Professorship of Ecological Services, Universitätsstraße 30, 95440 Bayreuth, Germany; 2 University of Wuerzburg, Department of Geography, Remote Sensing Unit, Am Hubland, 97074 Wuerzburg, Germany; 3 University of Bayreuth, Faculty of Biology, Chemistry and Earth Sciences, Professorship of Disturbance Ecology, Universitätsstraße 30, 95440 Bayreuth, Germany; Chinese Academy of Sciences, CHINA

## Abstract

The worldwide demand for food has been increasing due to the rapidly growing global population, and agricultural lands have increased in extent to produce more food crops. The pattern of cropland varies among different regions depending on the traditional knowledge of farmers and availability of uncultivated land. Satellite images can be used to map cropland in open areas but have limitations for detecting undergrowth inside forests. Classification results are often biased and need to be supplemented with field observations. Undercover cropland inside forests in the Bale Mountains of Ethiopia was assessed using field observed percentage cover of land use/land cover classes, and topographic and location parameters. The most influential factors were identified using Boosted Regression Trees and used to map undercover cropland area. Elevation, slope, easterly aspect, distance to settlements, and distance to national park were found to be the most influential factors determining undercover cropland area. When there is very high demand for growing food crops, constrained under restricted rights for clearing forest, cultivation could take place within forests as an undercover. Further research on the impact of undercover cropland on ecosystem services and challenges in sustainable management is thus essential.

## Introduction

Cropland expansion is one of the major anthropogenic factors causing loss of major natural ecosystems around the globe [1, 2]. With the increasing global population and demand for food, cropland continues to expand and has resulted in 'land grabbing' (large-scale acquisitions of agricultural land) mostly in developing countries of the tropics [3–5]. Vast areas of land in sub-Saharan African countries such as Sudan, Ethiopia and Kenya are leased to local and global investors for large scale agriculture [1, 6]. In most cases, local small-scale farmers are displaced when their land is needed for investment [7]. Thus, with growing population, poor technology and increasing 'land grabbing', local farmers in sub-Saharan Africa are often forced to look for unoccupied marginal lands in the mountains that are not optimal for growing crops due to extremely rugged topography. These ecosystems are generally threatened worldwide by land use/land cover (LULC) change and are under continuous pressure due to cropland expansion to feed the rapidly growing global population [7, 8]. Mountainous areas provide diverse ecosystem services such as water, sediment retention, erosion control, flood regulation and recreation, and they are hotspots of biodiversity [9–11]. In the long-term, conversion of forests and grasslands to croplands may lead to degradation of fragile mountainous ecosystems [12, 13]. This impact has been realized worldwide and protection of mountain ecosystems has gained attention [14–16]. However, in regions with slow industrial development and the majority of the population being subsistence farmers, the past efforts in protecting forests in mountainous areas only shifted the patterns and distribution of cropland instead of slowing its rate.

Agroforestry systems that combine multipurpose trees with crops have been effectively practiced in the past across different parts of the world. For instance, in India, wheat has been planted with *Eucalyptus* and *Poplar* plantation trees [17–21]. In the western Himalaya, rice and wheat are intercropped with tree species such as *Grewia optiva*, *Morus alba* and *Eucalyptus* [22]. In China, an area of over two million hectares was an intercrop of wheat and *Paulownia* trees during the 1990's [23]. Multipurpose trees such as *Ginkago biloba* have been grown in southern China with broad beans and wheat mixtures [24].

In Ethiopia, agroforestry systems (e.g. combining fruit trees, coffee, crops and vegetables as multi-story vegetation) have been traditionally practiced for hundreds of years [25]. However, this was mainly limited to flat and moderate slope areas of the southern and south western parts of Ethiopia. In the past, researchers have investigated the potential for the adoption of agroforestry systems in the mountainous areas of the Ethiopian highlands [25–27]. However, the transfer of agroforestry knowledge from the southern and southwestern regions to the mountainous regions of Ethiopia showed limited success. In most cases, tree-crop combinations in the mountainous areas of Ethiopia lasted only for short time until the forest land was fully converted to cropland. One example of such LULC patterns is undercover cropland inside forests in mountainous areas. In this study we define the term 'undercover cropland' as cultivation and growing of crops under forest canopies without future plans for transforming the forest into a multistory agroforestry system. To ensure sustainability of such complex systems where multistory vegetation types form an ecosystem, detailed analysis and assessment of its influential factors and emerging land management challenges is essential.

Remote sensing provides fast and reliable data for large scale assessment of vegetation cover. In principle, airborne and satellite remote sensing data are suitable for LULC classification [28–31]. However, due to their property of being a reflectance measure, these datasets are mainly based on canopy structure visible from above. Consequently undercover cropland cannot be fully detected. Most of the global land cover classifications in the past used coarse resolution data and provided only broad classes such as cropland-woodland and cropland-grassland mosaics. Thus, detailed analysis to capture the hidden cropland inside forests requires supplementing the remote sensing data with field surveying in order to obtain reliable results.

In this study, we assess undercover cropland area and its explanatory variables in the hilly terrains of the Bale Mountains of Ethiopia by analyzing field observed percent cover in combination with topographic and location factors using Boosted Regression Trees. The study is based on the hypothesis that topographic parameters such as slope, elevation and aspect as well as location factors such as distance to settlements and the national park influence undercover cropland inside forests in the Bale Mountains of Ethiopia. The main objectives are to i) map LULC and identify hotspots of cropland under forest canopies; ii) identify explanatory variables and map undercover cropland area; iii) assess the emerging challenges and future prospects of undercover cropland in the region.

## Materials and Methods

### Study site

**Fig 1:**
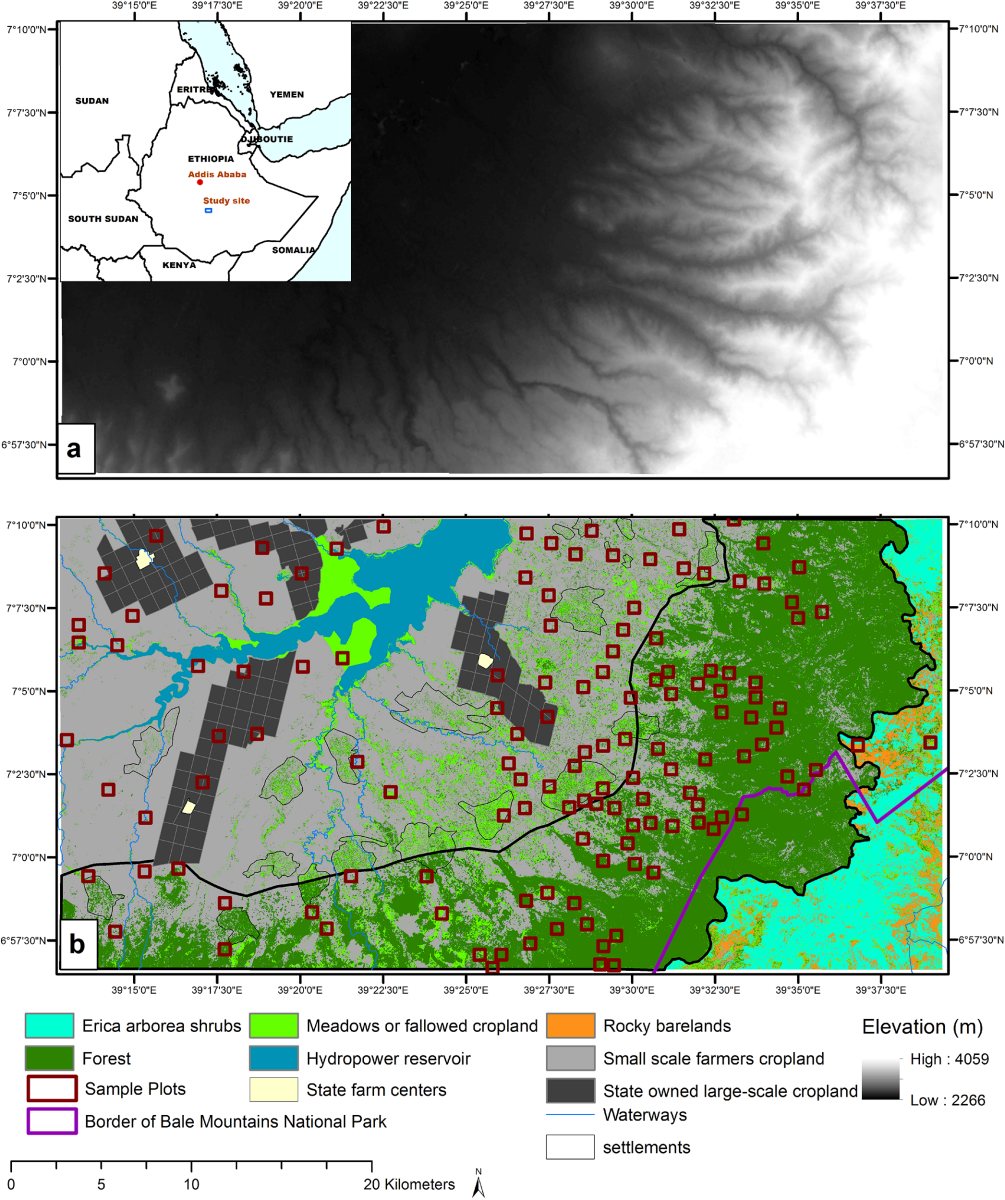
Study site and land use/land cover classes a) Location of the study site and distribution of sample plots b) Major land use/land cover types derived using Random Forest classification of RapidEye images. Field estimated percent cropland per plot is overlaid on the land use/land cover map.

Situated in the southeastern part of Ethiopia, the Bale Mountains are characterized by enormous ecological heterogeneity and steep gradients of altitudinal zones (Fig 1A). The site we selected for data sampling is part of the Adaba, Dodola, Asassa and Dinsho districts of the Arsi and Bale zones of the Oromia regional state of Ethiopia. It is adjacent to the boundary of Bale Mountains National Park (BMNP), which is known for its enormous biodiversity and insitu conservation of highly endangered mammals, birds, plants, and amphibians endemic to Ethiopia [32–35].

The region supplies diverse ecosystem goods and services to local and national beneficiaries. For instance, provisioning services dominant in the Bale Mountains and the surroundings include food, water, timber, fuelwood, and fodder. Regulatory services include flood regulation, erosion control, and water purification. Due to the availability of tourist attraction sites in the area, the aesthetic and recreational values are the other important services supplied by ecosystems in the Bale Mountains. The high population growth in the area has increased demand for food by local farmers, nearby villages, and towns. In the past, crop production in the area was concentrated in the lower escarpments and flat areas. However, with increased population the open grasslands and forest areas are nowadays intensively cultivated [36]. Furthermore, recent cropland expansion under forest canopies is becoming an ongoing practice. Local farmers expand croplands in the uplands where forest clearing is restricted by the local government. Such shifts in the patterns of land cover indicate potential for tradeoffs in the supplies of various ecosystem services such as provisioning and regulatory services.

The area is under continuous pressure from various actors and growing population in the surrounding districts, which pose a threat to conservation areas of the BMNP. The fact that the area is adjacent to the national park, situated at the border of the four districts mentioned above, and subject to varying altitudinal gradients makes it interesting for assessing the LULC, especially the patterns of cropland expansion.

### Remote Sensing data

In this study, RapidEye images [37] taken during dry periods (December 2012) were used for the LULC classification. The images used were orthorectified 3A products with the spectral bands: Blue (440–510 nm), Green (520–590 nm), Red (630–685 nm), Red Edge (690–730 nm) and Near Infrared (760–850 nm). Each image had a resolution of 5 meters and covered a total ground area of 625 km^2^ (image dimension of 25x25 m). The level 3A products are geometrically corrected for sensor-related effects using sensor telemetry. The bands are co-registered and spacecraft-related effects are corrected using attitude telemetry and best available ephemeris data. These products are further orthorectified using ground control points (GCPs) and Digital Elevation Models (DEMs).

The two scenes were mosaicked using the ENVI 5.0 [38] georeferenced mosaicking function. Atmospheric and topographic corrections were performed using ATCOR 3 software [39]. ATCOR 3 was preferred used for our largely mountainous study site as it provides algorithms for correcting images taken from rugged topography [39, 40]. ATCOR 3 corrects changes in spectral reflectance of objects, removes haze and reduces the effects of shadow in mountainous terrains [39, 40].

### Field data sampling

**Fig 2:**
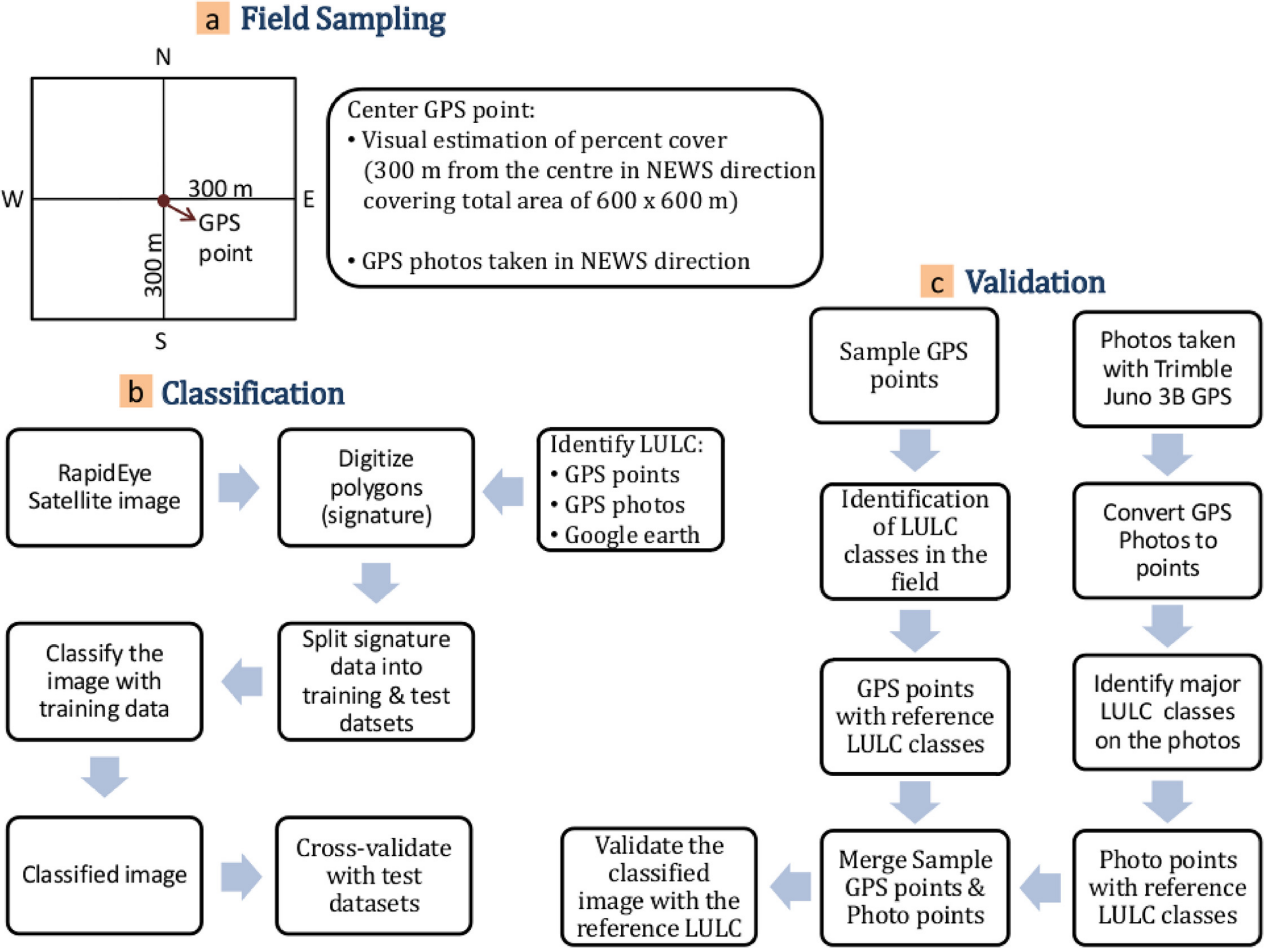
General workflow of a) Field data sampling b) Image classification c) Validation of classification results.

A field visit in the study site was carried out between October and December 2012, the same season in which the RapidEye satellite images were taken. Land use/land cover related data was collected with the official permission of the Adaba, Dodola, Asassa and Dinsho districts of Ethiopia, given by the local village leaders and private land owners in the area. Since part of the site is inside the boundary of the Bale Mountains National Park, permission to collect land cover related data was granted by the head of the national park. The study does not involve animals in experiments and we confirm that the field studies did not involve endangered or protected species. A total of 136 sample plots were laid out randomly at varying intervals based on heterogeneity of LULC and accessibility of the landscape (see Fig 2 for details of steps in field sampling). The interval between the sample plots was long (up to 5 kilometres) in homogenous areas while it was short (1–2 kilometres) in heterogeneous landscapes. The sample plots were laid out with a distance of 300 m from the center point in four directions: North, East, West and South with each having an area of 0.36 km^2^ (Fig 2A). The sampling of data was carried out for each plot and recorded in the worksheets prepared for field surveying.

Trimble Juno-3B GPS was used to measure the coordinates at the centre of the sample plots to identify the spatial location where the data was recorded. Percentage cover of different LULC classes was estimated for each plot taking the GPS centre point as a reference. The percent cover for each LULC class was estimated visually in a range of 0 to 100. The visual estimation was made relative to the total area of the plot i.e. 0.36 km^2^. For this, discontinuous LULC classes (e.g. forest patches, meadows, croplands) were assumed to be as continuous relative to the plot area to assign total percent cover per plot. As a visual guideline the area covered by 1% of the LULC class as a percentage of 0.36 km^2^ which is equivalent to 0.0036 km^2^. Thus, one percent cover of LULC is equivalent to a square of 0.06 x 0.06 km. Besides the percentage cover per plot, LULC classes at the GPS points were recorded for later use in the validation of classification results. To further support validation of results from the satellite image classification, photographs were also taken in four directions from the GPS centre point using the built-in Trimble Juno 3B camera.

### Land use/land cover classification

The Random Forest (RF) classifier [41] implemented in R 3.02 statistical software package (R Development Core Team 2008)was used to classify the RapidEye images in order to derive LULC classes. This method uses an ensemble of tree-like classifiers similar to Bagging Trees in which bootstrap samples are drawn to construct multiple trees [41]. RF is a refinement of Bagging Trees, since it improves bagging by “de-correlating” the trees. In the RF methodology a large number of trees (500 to 2,000) are grown. Unlike Bagging Trees, in RF each tree is grown with a randomized subset of predictors from which the name random forests is derived. Trees are grown to maximum size without pruning, and are aggregated by averaging to select only the best split among a random subset at each node [41].

Random Forest classifier searches a random subset of features from the total number of predictors to find the best split at each tree node in order to minimize the correlation between classifiers in the ensemble. The classifier ensembles are based on the concept that a set of classifiers performs better than individual classifiers [42]. Since the resampling is not based on weighting, the RF classification method is not sensitive to noise or overtraining [43]. Moreover, the method has been widely used for classification since it provides high classification accuracy [43–46].

For the purpose of classification, eleven major LULC classes (Table 1) were identified based on field observations of the study site. Accordingly, sample polygons (ESRI shape files) representing each class were digitized based on LULC data collected at 136 field sample plots and georeferenced GPS photographs taken at the center of the plots. For each LULC class, 250 samples (pixels) were randomly extracted from the list of image features (S1 Fig) and split into training (63%) and testing (37%) datasets. Each tree is grown on the training datasets using the bootstrap sampling process. The number of trees in the forest (*n*_*tree*_) and the number of random subsets of features tried at each node (*m*_*try*_) were set to 1000 and 3, respectively. For the LULC classification, the five bands and indices calculated from the RapidEye images were evaluated for their importance in identifying different LULC classes. In RF, the importance of a feature is determined based on how often the feature was used in the tree construction process. S1 Fig illustrates the relative importance of the features evaluated for the RF classification showing the most used and least used features.

**Table 1:** Description and broad categories of the land cover classes.

LULC_ID	Description of classes	Broad category
1	Dense forest	Forest
2	Single scattered trees	Forest
3	Recently grown green crops	Cropland
4	Close to ripening green crops	Cropland
5	Ripened crops or recently harvested land	Cropland
6	Cultivated land_dark colored wet soils	Cropland
7	Cultivated land_grey colored dry soils	Cropland
8	Meadows and fallowed croplands	Meadows or fallowed croplands
9	Reservoirs	Reservoirs
10	Shrublands	Erica arborea shrubs
11	Bare rocks	Rocky barelands

After classification into the eleven classes, similar LULC types were grouped into major classes such as cropland, forest, meadows or fallowed cropland, shrubs and barelands. From classes listed in Table 1, classes 1(dense forest) and 2 (single scattered trees) were grouped into forest since they represent different types of forest. Similarly, classes 3 to 7 were grouped as cropland because they all represent areas of land used for growing crops. The boundaries of state farms and major settlement areas were digitized manually from the RapidEye images from high resolution Google Earth images and GPS points recorded during the field observation and overlaid on the produced LULC map.

### Validation

For validation of the RF classification, as a cross-validation step, error measurements used were of twofold: errors calculated from the Out-Of-Bag (OOB) subset of data and those calculated from the test data subset. Firstly, performance of the RF classifier was evaluated using cross-validation during the model training process. Each tree is grown using a bootstrapping technique that involves sampling in which some of the data are left out i.e. the Out-Of-Bag (OOB) sample, while some others are repeated in the sample [41]. In the training process, 2/3 of the training data was actually used for tree construction leaving 1/3 as OOB. Since the OOB data was not used for tree construction, in parallel with the training step, the OOB samples were used for cross-validation. Secondly, independent test datasets were used to assess the performance of RF algorithm by calculating the proportion of test elements that were incorrectly predicted. Finally, confusion matrices were calculated for each predicted class and its corresponding reference class as well as the OOB and test error rates. The steps in the cross-validation of the classification result are shown in Fig 2B.

Besides the cross-validation step, reference LULC classes collected at the center of the sample plots were used in combination with reference LULC classes identified from GPS photos taken in North, East, West and South directions (Fig 2A). The GPS photos were converted to points using QGIS 2.0.1 software and the reference LULC classes were assigned to the points based on the photos. The sample GPS points were merged with the photo points and were used to validate the major LULC such as cropland, forest and meadows or fallowed croplands (Fig 2C). A total of 611 (120 GPS points taken at the center of the plot and 491 points extracted from GPS photos taken in four directions from the center point) were used for the accuracy assessment.

### Identifying factors influencing undercover cropland area

Boosted Regression Trees (BRTs) [47], an ensemble method used for fitting statistical models was used to identify influential variables for cropland area. At each sample plot, undercover cropland area was calculated as the product of area of the sample plot and field estimated percent undercover cropland. Boosted Regression Trees combine algorithms of regression trees that use recursive binary splits to relate a response to their predictors and boosting that combines simple models to improve predictive performance [47–49]. Moreover, BRTs are preferred since they capture complex structures that arise from spatial autocorrelation within a dataset. In contrast, with simpler modeling approaches, results are highly influenced by spatial autocorrelation in datasets [50]. BRTs is an additive regression model that undergoes stagewise fitting without changing existing trees when the model enlarges [49].

Boosted Regression Trees were constructed using R 3.02 statistical software package [51] to identify the relationship between undercover cropland area and potential influential factors. The factors considered include elevation, slope, east aspect, west aspect, north aspect, south aspect, distance to the national park, and distance to settlement areas. Slope and aspect maps were derived from digital elevation model, while distance to the national park and distance to settlements were calculated from sample plot centers to the boundaries of the national park and settlement areas. These factors were selected for analysis since they often dictate agricultural activities in mountainous regions [52, 53]. The four aspect raster layers were extracted from aspect map as dummy values of 0 and 1. All the raster layers were rescaled to a grid size of 10 pixels. Values of all the influential factors were calculated for the sample plots where undercover cropland area was estimated. The BRTs model was set with Gaussian error distribution, tree complexity of 2, learning rate of 0.008 and bag fraction of 0.75 while the maximum number of regression trees specified was 3000. The results of the relationship between the influential factors and undercover cropland area estimated in the field were plotted and visualized as fitted functions showing the relative importance of each influential factor as a percentage. In addition to the undercover cropland inside forests, influential variables were predicted for total cropland area per plot both from field estimated data and the RapidEye images for further understanding of factors determining the general patterns of cropland expansion in the region. See [47] for details about Boosted Regression Trees and the model settings.

### Prediction and mapping of the undercover cropland

The raster layers of influential variables tested using BRTs model in section 2.6 were used to predict and map the percentage cover of undercover cropland. Field estimated percentage undercover cropland data from 135 sample plots were used to predict the undercover cropland from the most influential factors such as elevation, slope and east aspect using Boosted Regression Trees. To obtain reliable result, the prediction was done using 16-fold cross-validation of the data. Hence, the final predicted undercover map is the result of the 16-fold cross-validation. Furthermore, the predicted map of undercover cropland was overlaid with the forest layer derived from the classification of RapidEye images to produce the final map of undercover cropland.

## Results

### Land use/cover types

The results of the RF LULC classification are presented in Fig 1B. The major LULC types in the study area include small-scale croplands, state owned large-scale croplands, forest land and meadows. The forests are situated in the upper escarpments of the area while croplands mainly occupy the lower escarpments. The upper most escarpments adjacent to the forest land are *Erica arborea* shrublands. These shrublands are found mixed with rocky barelands in extremely rugged topography. Settlement areas consist of built-up areas mixed with croplands, meadows or fallowed croplands, and scattered trees. In the study site, there is a large reservoir that belongs to Melka Wakena hydropower station and is used by the government of Ethiopia to generate electricity. The area is near the BMNP and cropland has extended to parts of the national park shrinking the area which was previously part of the national park (Fig 1B).

### Accuracy of classification

The OOB and test error rates of the RF classification for each LULC class are presented as confusion matrices in Tables 2 and 3 respectively. Most of the LULC types were classified with an OOB error rate of 4.87% and test set error rate of 4.22%. The error rate in each class shows the proportion of misclassified observations in that class, whereas the average test error rate shows the proportion of misclassified observation for the entire dataset. The mean test error rate per class ranges from 0 to 10.77.

**Table 2:** Confusion matrix showing the error rate of the training dataset (OOB error rate).

Predicted													
LULC_ID		1	2	3	4	5	6	7	8	9	10	11	Class error
Reference	1	121	7	0	0	0	0	0	0	0	0	0	0.05
	2	4	124	5	5	0	1	0	0	0	0	0	0.11
	3	0	2	108	1	0	0	3	6	0	0	0	0.10
	4	0	2	0	120	0	0	0	0	0	0	0	0.02
	5	0	0	0	0	123	0	0	0	0	0	2	0.02
	6	0	0	0	0	1	123	1	0	0	0	0	0.01
	7	0	0	1	0	0	1	111	0	0	0	0	0.02
	8	0	0	1	1	0	0	0	130	0	0	0	0.02
	9	0	0	0	0	0	0	0	0	121	1	0	0.01
	10	0	0	0	0	3	0	0	0	1	117	9	0.08
	11	0	0	0	1		3	1	0	0	5	107	0.11

Average OOB estimate of the error: 4.87%.

**Table 3:** Confusion matrix showing the error rate of the test dataset.

Predicted													
LULC_ID		1	2	3	4	5	6	7	8	9	10	11	Class error
Reference	1	113	9	0	0	0	0	0	0	0	0	0	0.07
	2	5	101	3	1	0	0	1	0	0	0	0	0.09
	3	0	11	116	1	0	0	0	2	0	0	0	0.11
	4	0	0	0	128	0	0	0	0	0	0	0	0.00
	5	0	0	0	0	119	0	0	0	0	0	3	0.02
	6	0	0	0	0	0	125	1	0	0	0	0	0.01
	7	0	0	0	0	0	0	137	0	0	0	0	0.00
	8	0	0	1	1	0	0	0	116	0	0	0	0.02
	9	0	0	0	0	0	0	0	0	128	0	0	0.00
	10	0	0	0	0	0	1	0	0	0	118	4	0.04
	11	0	1	1	0	4	2	0	1	0	5	116	0.11

Average estimate of the error rate for the test dataset: 4.22%.

The OOB error value computed from the left out data during the training process showed a very good performance of the RF model with an average model accuracy of 95%. The calculated test set errors imply that all the LULC types were classified with an average accuracy above 95%. The numbers in the matrix show the number of samples from the actual class (reference class) that is correctly or wrongly predicted. For instance, in the training set, class 1 has 128 samples of which 121 (94%) are correctly classified while 7 (6%) are misclassified as class 2. In the test set, class 1 has 122 samples of which the 113 (92%) are correctly classified and 9 (8%) samples are misclassified as class 2. The values for the rest of the classes in confusion matrices (Tables 2 and 3) can be also interpreted in a similar way.

The matrices indicate major confusion between the “dense forest” and “single scattered tree” classes, but also between the class “single scattered trees” and “recently grown green crops”. These overlaps are very well known and can be attributed to the spectral similarity among green vegetation classes. However, the high resolution of RapidEye data may be one reason that mixed pixel problems have less influence on classification accuracies as observed also in classification of Savannah landscapes of West Africa using ASTER 15 m datasets [30]. Minor confusion between “*Erica arborea* shrubs” and “bare rocks” can be explained by the topography, because both classes mainly occur in rugged terrain where shadows occur which can cause uncertainty during classification.

The validation results for the three major categories of LULC classes (Cropland, forest, meadows or fallowed croplands) are presented in Table 4. These LULC were considered for further validation besides the cross-validation step since they are the central focus of the research on the undercover cropland.

**Table 4:** Accuracy assessment of classification results for dominant land cover classes in the study site.

		Ground truth points				
	LU_Name	Croplands	Forests	Meadows or fallowed croplands	Total	Class accuracy (%)
Predicted	Croplands	**379**	8	16	403	94.04
	Forests	8	**111**	6	125	88.80
	Meadows or fallowed croplands	4	8	**71**	83	85.54
	Total	391	127	93	611	
	Sum of correct predictions				561	
	Overall Accuracy				91.82	
	Kappa Coefficient				0.84	

Cropland and forest were classified with accuracy of 94% and 89% respectively. The accuracy of classification for the class meadows or fallowed croplands was 86%. The overall accuracy achieved for the three major LULC classes was 92% with a Kappa coefficient of 0.84 (Table 4). In general, the cross-validation and validation results showed that the RF classification was performed with an acceptable accuracy.

### Relative importance of factors influencing undercover cropland area

**Fig 3:**
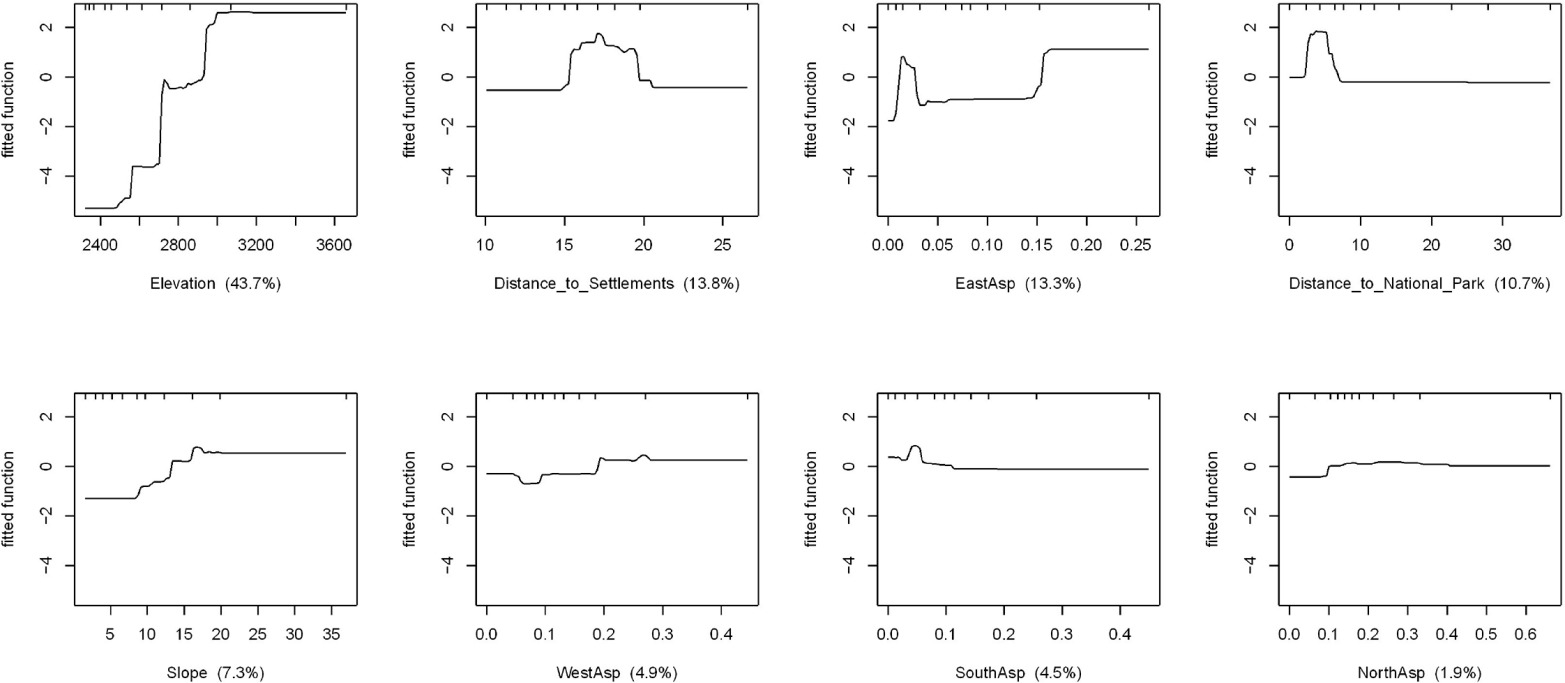
Boosted Regression Trees fitted model showing the relative importance of influential factors of undercover cropland area calculated from field estimated percent cover.

The Boosted Regression Trees fitted model for each of the influential factors of undercover cropland area calculated from field estimated percent cover is presented in Fig 3. The area of undercover cropland rises with increasing elevation, slope, and distance to major settlements while it decreases with distance from the national park. However, after certain limit the graph remains constant with a value of 0 showing that there is no undercover cropland above such limits. Similar patterns of the relationships between aspect and undercover cropland area were observed (Fig 3).

The results of the BRTs model of undercover cropland area showing the relative influence of the variables considered and predictive performance of the model are summarized in Table 5. The Boosted Regression Trees model for area of undercover cropland calculated from field estimated percent cover can explain 70.35% of the total deviance. The deviance was determined using a Gaussian family distribution function and is a measure equivalent to R^2^.

**Table 5:** Influential factors of undercover cropland area (ha) calculated from field estimated percent cover derived using BRTs model with tree complexity, *tc* of 2, learning rate, *lr* of 0.008 and bag fraction, *bf* of 0.75.

Explanatory variables		Relative influence (%)	Rank
Elevation		43.7	1
Distance to Settlements		13.80	2
East aspect		13.30	3
Distance to National Park		10.70	4
Slope		7.30	5
West aspect		4.90	6
South aspect		4.50	7
North aspect		1.90	8
**Predictive performance of the BRTs model**			
Mean total deviance	Mean residual deviance	Estimated CV Deviance	CV correlation
41.25	12.23	26.94 (SE ± 4.5)	0.59 (SE ± 0.05)

Elevation is the most influential factor accounting for 43.70% of the variance explained. Distance to settlements and east aspect consist of 13.80 and 13.30% respectively. The percentage of explained deviance by distance to national park is 10.70%, while slope consists of about 7.3% of the total explained deviance. West aspect, south aspect and north aspect each explained only less than 5% of the deviance (see Fig 3).

Besides the undercover cropland, for comparison, the BRTs results of cropland area and its influential factors is provided in the electronic supplementary information. Cropland area here refers to the area of land in sample plots where crops are grown i.e. including the area of cropland estimated as an undercover and the area of cropland in open areas. S2 Fig shows BRTs fitted model for influential factors of cropland area calculated from plot-level field estimated percent cover. The proportion of total deviance explained by slope, elevation and distance to national park are 34.7, 30 and 19.7% respectively while aspect appears less influential for cropland area estimated at sample points (S2 Fig). The BRTs fitted models for plot-level cropland area calculated from RapidEye images (S3 Fig) show that elevation is most influential factor accounting for 57.7% of the total explained deviance. Due to the difficulty of calculating undercover cropland from RapidEye images, the calculated cropland area here refers to what can be captured with the images without including the undercover cropland. Distance to national park and distance to settlements each account for 8.7% of the deviance. Aspect appears less influential when compared with the aforementioned parameters (S3 Fig). For details on the relationship between the holdout deviance and the number of trees in BRTs model for the undercover cropland area calculated from field estimated percentage cover and RapidEye images, refer to S4 Fig

### Predicted undercover cropland

**Fig 4:**
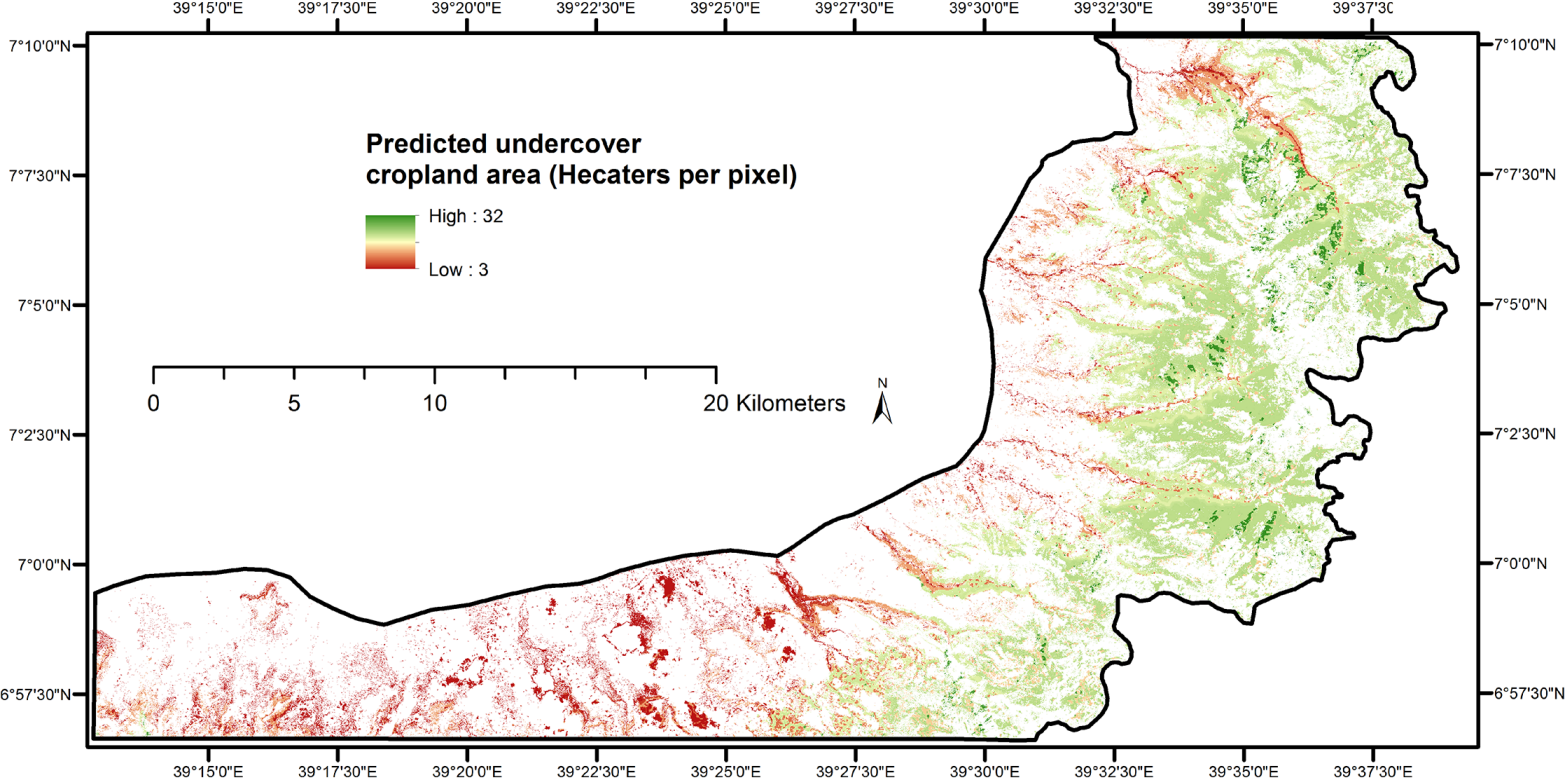
Undercover cropland area predicted from most influential topographic factors identified using Boosted Regression Trees.

The land use in the study site includes cropland inside the remnants of forests. Though the classification of RapidEye images shows forest as dominant LULC type in the upstream areas, there is cropland under the tree canopies. The undercover cropland area predicted using only the most influential variables slope, elevation and east aspect ranges from 0 to 32 hectares per pixel (Fig a in S5 Fig). Undercover cropland predicted from all the topographic variables (elevation, slope, east aspect, west aspect, north aspect and south aspect) using BRTs is presented in (Fig b in S5 Fig). Probability of undercover cropland in the area ranges from 0 to 25 hectares per pixel when all topographic factors are included in the prediction. S6 Fig compares undercover cropland area predicted using the selected variables slope, elevation and east aspect (Fig a in S5 Fig) with all variables (Fig b in S5 Fig). The scatter plot showed that there is no much difference between undercover cropland area predicted using only the most influential variables and all the variables. S7 Fig presents the comparison of cropland area calculated from RapidEye images and area estimated in the field. In this study, we assumed that remote sensing underestimates area of cropland since undercover cropland cannot be detected. However, results at some of the sample plots showed that the classification of RapidEye images overestimated cropland area which might be due to error in classification of LULC classes such as meadows as croplands (S6 Fig). Although classification of remote sensing images provides useful information, it is usually subject to unavoidable uncertainties that call for more research in this discipline. The undercover cropland map produced by overlaying the probability map with the forest layer from RapidEye image classification is shown in Fig 4.

**Fig 5:**
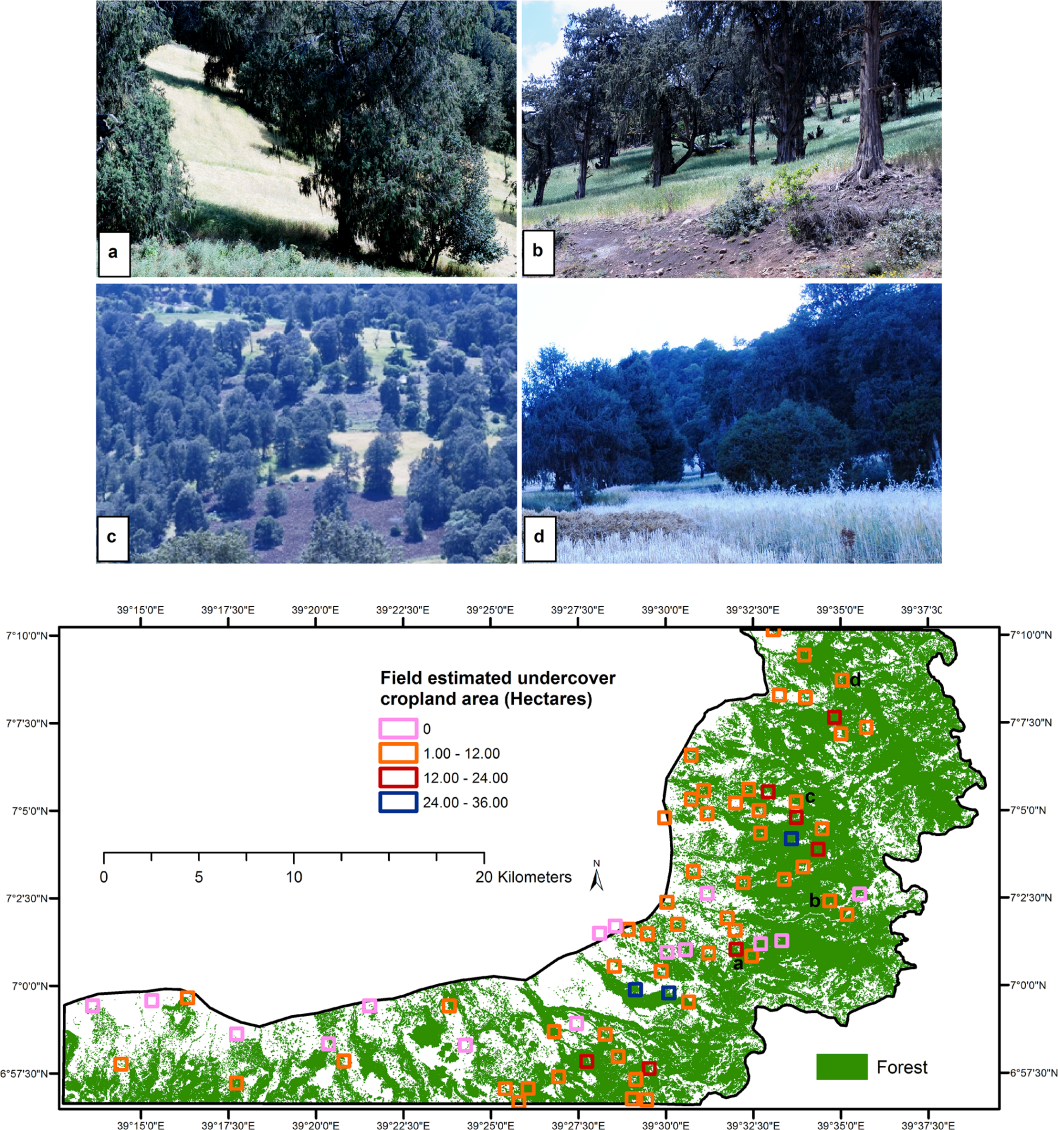
Undercover cropland area calculated from field estimated percent cover for selected sample plots with an area of 36 hectares. Photos labeled *a* to *d* show close range view of the undercover cropland taken at the location on the map labeled with the same letters.

Undercover cropland is located in the upper escarpments though there is no undercover cropland in the upper most extremes of the site (Fig 4). The upper most extremes are dominated with dense *Erica arborea* shrubs and are not suitable to grow crops due to the extremely cold climatic condition and also the dense shrubs that require complete clearing for growing crops. Thus, the larger portion of undercover cropland forms a belt in the upper escarpments of the study site (Fig 4). Field observations confirmed that this belt is dominated with *Juniperus procera* trees. The undercover cropland forms a vertical stratum with cropland as undergrowth and trees being the upper canopy (Fig 5). As it was observed in the field, the major crop cultivated inside the forest is wheat. The reasons for this preference shown by farmers are most likely the high market demand for wheat and provision of improved seeds and fertilizer by the government due to its recent plan to improve crop production.

The photos in Fig 5 show a close-range view of undercover cropland inside forests from sample plots. They reveal hidden cropland inside the areas that were classified as forest using the RapidEye images which mainly capture the view of forest canopy from above. Cropland was observed inside forests including very steep terrain areas that were entirely covered with forest and/or with semi-open woody pasturelands that were previously used for livestock grazing.

## Discussion

### Influential factors of undercover cropland

Growing of food crops in the Bale Mountains involves not only cultivation of open lands with moderate slopes but also cultivation inside forests in the upper escarpments. The findings of this study confirmed that topographic parameters such as elevation, slope and aspect are important factors that influence cropland area in mountainous regions. These parameters were found also important determinants of soil properties and vegetation types in the Bale Mountains [54, 55]. Similar studies in mountainous areas also demonstrated that elevation, slope and aspect are among the influential factors that need to be considered in mountain ecosystem conservation and habitat management [8, 56–58].

In the Bale Mountains, there is difference in the impacts of topographic parameters on cropland area as a whole and the area of cropland which is cultivated as an undercover. For instance, inverse relationship was observed between cropland area and topographic parameters such as elevation above sea level and slope (in degrees) which conforms to the findings in other mountainous areas [59]. However, area of cropland cultivated as an undercover increased with increasing elevation and slope inside semi-protected forests. The extremely steep terrains correspond to high elevation areas that were not suitable for growing cereal crops. Nevertheless, recently farmers started growing crops in these areas which is an indication of impacts of climate change in the region. Undercover cropland is influenced also by topographic aspects that modify microclimatic conditions. Easterly aspect was found to be more determinant compared with other topographic aspects. The main reason for this may be the exposure of land under forest canopies to morning sunlight which provides favorable conditions for vegetation growth inside forests [60].

Besides the topographic parameters, the extent of undercover cropland was found to be influenced by location parameters such as distance to national park and settlement areas. There is a positive relationship between cropland area and distance to the national park whereas the area of cropland cultivated as an undercover showed inverse relationship with distance to national park. An inverse relationship might result from the fact that areas close to national parks are relatively protected and hence, farmers’ only option has become growing crops as an undercover. Cropland area in general increases close to settlement areas but area of cropland cultivated as an undercover inside forests decreases near major settlement areas.

Generally, the study indicated the major topographic and location parameters that limit human activities regarding growing of food crops in a mountainous region. The pattern of growing crops as an undercover inside forests in the mountain escarpments could have series consequences on ecosystem services which demand prompt solutions to address the ongoing challenge in the region. The potential consequences on ecosystem services and the challenges of growing crops as undercover are discussed in sections 4.2 and 4.3 below.

### Consequences on ecosystem services

The study site we investigated supplies diverse ecosystem services that are essential for the livelihood of the local people. The undercover cropland in this mountainous region thus has multifaceted impacts on supplies of these ecosystem services. For instance, the forest in the area is the major source of timber and fuelwood for the local people and to markets in the surroundings. However, the recent undercover growing of crops inside the forest in the upper steep slope areas gradually degrades the forest thereby reducing supplies of timber and fuelwood. Moreover, most of the forested areas where undercover cropland is maintained were previously mixed with pasture. Hence, cultivation of cropland as an undercover may decrease the supply of fodder for livestock, indirectly reducing the livestock production [61, 62].

Cropland under forest canopies often exposes soil to erosion, especially in the extremely steep terrains [63, 64]. In addition, land degradation is likely to happen as more fertile soil is washed away. This may in turn damage the agricultural prospects as well as the supplies of other ecosystem services in the area [65]. As it was observed during the field surveying, soil eroded from the upstream area of the Bale Mountains has already been a threat to the supply of electricity by the local hydropower, Melka Wakena, due to the deposition of sediments in the reservoir. During the rainy season the hydropower reservoir is usually filled with sediments coming from the upstream area. Sediment deposition in the reservoir increases the cost for cleaning and maintenance of the reservoir if undercover cropland continues without considering preservation of forests in the upstream areas. Increased runoff from the upstream areas and sediment deposition in the rivers and reservoir affects also the supply of clean water to nearby beneficiaries [7, 66, 67].

### Challenges in transforming an undercover cropland into a sustainable agroforestry system

Conversion of open forests with a herbaceous layer to undercover cropland inside forests in the Bale Mountains cannot be regarded as a sustainable agroforestry system. Undercover cropland lacks the following major features of an agroforestry system that need to be addressed.

#### Selection of tree-crop combination

An agro-forestry system involves multistory land use and is widely acknowledged for its capacity to ensure sustainable land use and resource management [68, 69]. Traditional agroforestry is usually a multistory composition of selected multipurpose trees, crops like maize, fruit trees, and vegetables [70, 71]. Agroforestry systems can be used as a remedy for severe environmental problems in highland areas but require selection of proper species of trees and crops [72–75]. Unlike traditional agroforestry systems involving planned selection and growing of tree-crop combinations, the undercover cropland is intended mainly for expanding cropland area without proper selection of tree-crop combination.

#### Management of trees and crops

In a well-managed agroforestry system, growing crops like wheat under tree canopies (e.g. Eucalyptus) provides favorable microclimatic conditions for the crops [17, 76, 77]. Tree canopy structure and distance from crops affects crop performance [21, 19, 23]. In some cases, toxic effects from trees could reduce the crop yield [18, 20]. Management practices in an agroforestry system depend on the crops growing under the canopies [78]. Considering the aforementioned typical features of tree-crop combinations, the undercover cropland observed lacks proper management of trees and crops as a multistory land use since the main goal of the farmers is to find a land for growing food crops.

#### Farmers' lack of experience in agroforestry

In the Bale Mountains, traditional knowledge of agroforestry is limited because the people in the area are mainly used to monocropping and livestock production. Thus, the undercover cropland introduced in the area brings forth dilemmas as to whether the farmers are adopting the knowledge of agroforestry from other parts of the country or using it as an opportunity for clearing forests to expand cropland in unoccupied forest land restricted by the government. It was introduced by the local farmers as a strategy for acquiring more land for growing crops inside remnants of the forest in the uplands where clearing of forest is restricted by the government. The system emerged as a result of the desperate need for food crop production and resembles a traditional agroforestry system because it involves growing of crops under tree canopies.

In general, given its current status, the undercover cropland would not eventually develop to an agroforestry system and it will only aggravate forest degradation. Thus, it can only be considered as an initial stage of deforestation which will later end up in complete clearing of the forests by gradually decreasing the forest density [79]. The use of open forests for livestock production instead of undercover cropland might be a more sustainable land use. For instance, it is a very well established traditional land use, e.g., in the European Alps where it is known as wood pasture [80].

## Conclusions

Although satellite image classification can be used for LULC mapping, assessment of undercover cropland inside forests requires detailed field surveying. The study confirmed that analysis of field observed percent cover and topographic parameters such as elevation, slope and aspect using Boosted Regression Trees enables assessment of undercover croplands in forested areas which otherwise are not detectable with remote sensing data. The findings showed that the extent of undercover cropland is determined by elevation, slope, aspect, distance to national park and distance to settlements. Among the topographic parameters elevation, slope and east aspect were found to be the most influential factors for growing of crops inside forests, having a positive relationship with undercover cropland area. Similarly, distance to settlements showed a positive relationship with undercover cropland area while an inverse relationship was observed with distance to national park. Therefore, while planning land use and ecosystem management in mountainous regions, decision-makers should take into account the relative importance of these parameters.

Further research is essential to find methods for designing and implementing policies that ensure sustainable supplies of ecosystem services and nature conservation such as Payments for Ecosystem Services (PES) schemes. Stakeholders such as farmers, government officials, environmental authorities, hydropower company, and the Bale Mountains National Parks (BMNP) should be incorporated into decision making. Further research is also needed to create awareness and find alternative solutions for the livelihood of the local people to reduce the expansion of cropland in extremely steep areas of this mountainous region. Since the undercover cropland observed resembles a traditional agroforestry system, there is potential to transform it in to a more sustainable agroforestry system. The following points should be considered to develop an undercover cropland into a sustainable agroforestry system. Firstly, proper selection of tree-crop combinations that are suitable for mountainous areas needs to be identified and used by the farmers in the area. Secondly, management practices for trees and crops in the undercover cropland areas should aim at ensuring sustainable supplies of ecosystem services. Lastly, knowledge transfer from well-established agroforestry areas to the mountainous undercover cropland areas is essential.

## Supporting Information

S1 Fig Relative importance of features used for classification of RapidEye images using RF classification. (PDF)

S2 Fig Boosted Regression Trees (BRTs) fitted model showing relative importance of influential factors of cropland area calculated from field estimated percent cover. (PDF)

S3 Fig Boosted Regression Trees (BRTs) fitted model showing relative importance of influential factors of cropland area calculated from RapidEye images. (PDF)

S4 Fig Number of trees vs total holdout deviance. Plots created using undercover cropland area calculated from field estimated percent cover (**Fig a**). Plots created using cropland area calculated from field estimated percent cover (**Fig b**). Plots created using cropland area calculated from RapidEye images (**Fig c**).
(PDF)

S5 Fig Predicted undercover cropland in hectares per pixel. Prediction using only most influential factors slope, elevation and east aspect (**Fig a**). Prediction using all topographic factors slope, elevation, east aspect, west aspect, south aspect and north aspect (**Fig b**).
(PDF)

S6 Fig Comparison of undercover cropland area (hectares) predicted from X, all variables slope, elevation, and aspects (east, west, south and north) with Y, only most influential variables (slope, elevation and east aspect). (PDF)

S7 Fig Comparison of cropland area (hectares) X, estimated in the field and Y, cropland area calculated from RapidEye images. (PDF)

S1 File Datasets used for the study. (ZIP)
