# Correction: Unveiling Undercover Cropland Inside Forests Using Landscape Variables: A Supplement to Remote Sensing Image Classification

**DOI:** 10.1371/journal.pone.0137150

**Published:** 2015-08-25

**Authors:** Yohannes Ayanu, Christopher Conrad, Anke Jentsch, Thomas Koellner

There are errors in the first paragraph of the “Predicted undercover cropland” section of the Results. “Hectares per pixel” should read “m^2^ per pixel.”

There are errors in Fig 4 and in its caption. Please see the complete, correct Fig 4 here.

**Fig 4:**
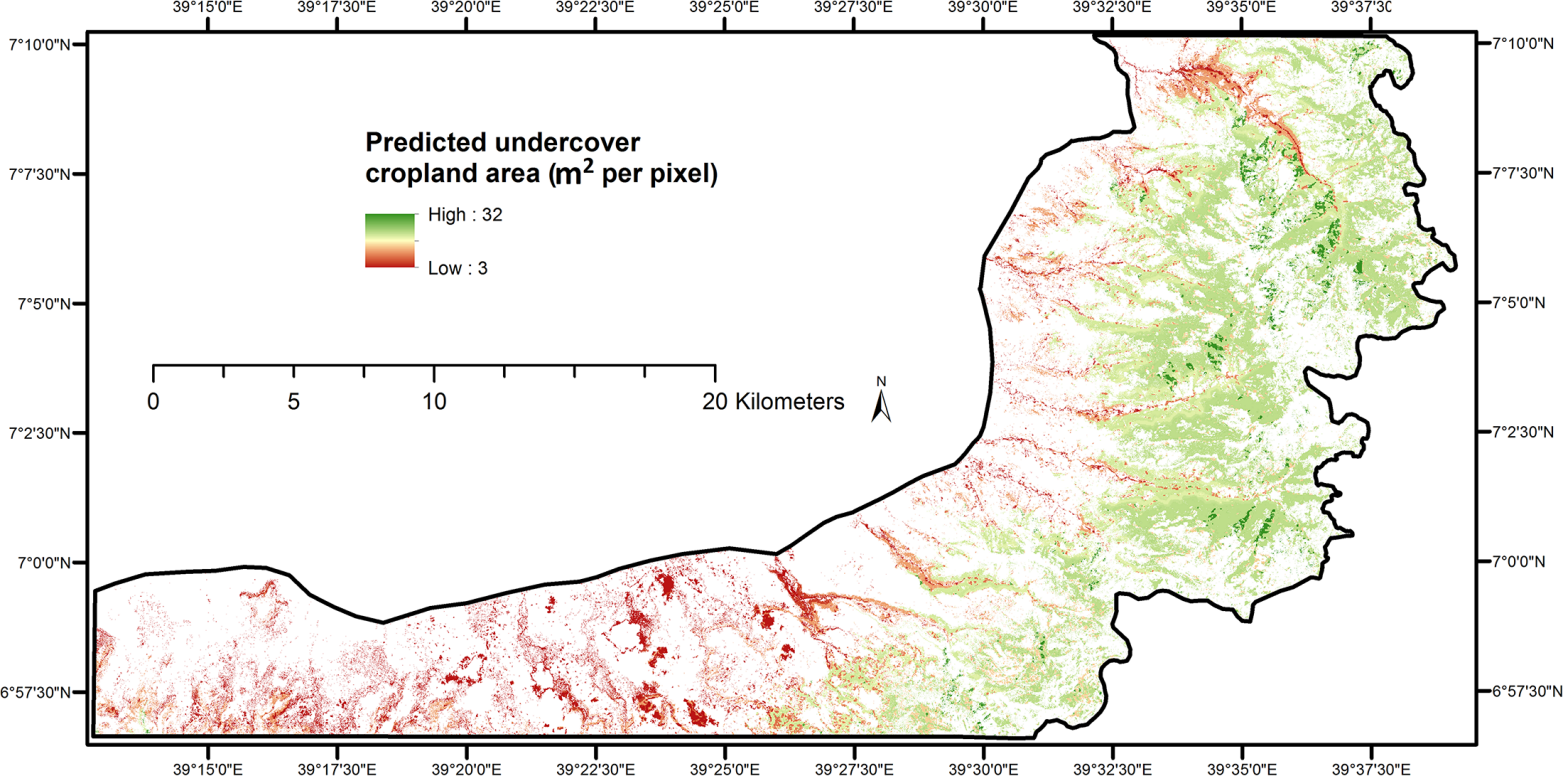
Undercover cropland area predicted from most influential topographic factors identified using Boosted Regression Trees. (pixel size of 100 m^2^)

There are errors in the caption for S5 Fig. Please view the correct S5 Fig. caption below.

## Supporting Information

S5 Fig Predicted undercover cropland in m^2^ per pixel. Prediction using only most influential factors slope, elevation and east aspect (**Fig a**). Prediction using all topographic factors slope, elevation, east aspect, west aspect, south aspect and north aspect (**Fig b**). Pixel size is 100 m^2^.
(PDF)
