# Supplementary material for: Unveiling Undercover Cropland Inside Forests Using Landscape Variables: A Supplement to Remote Sensing Image Classification
Source: PLoS One. 2015 Jun 22;10(6):e0130079. doi: 10.1371/journal.pone.0130079 (PMC4476797; doi:10.1371/journal.pone.0130079)
Supplement: S1 Fig — (PDF) [file pone.0130079.s001.pdf]

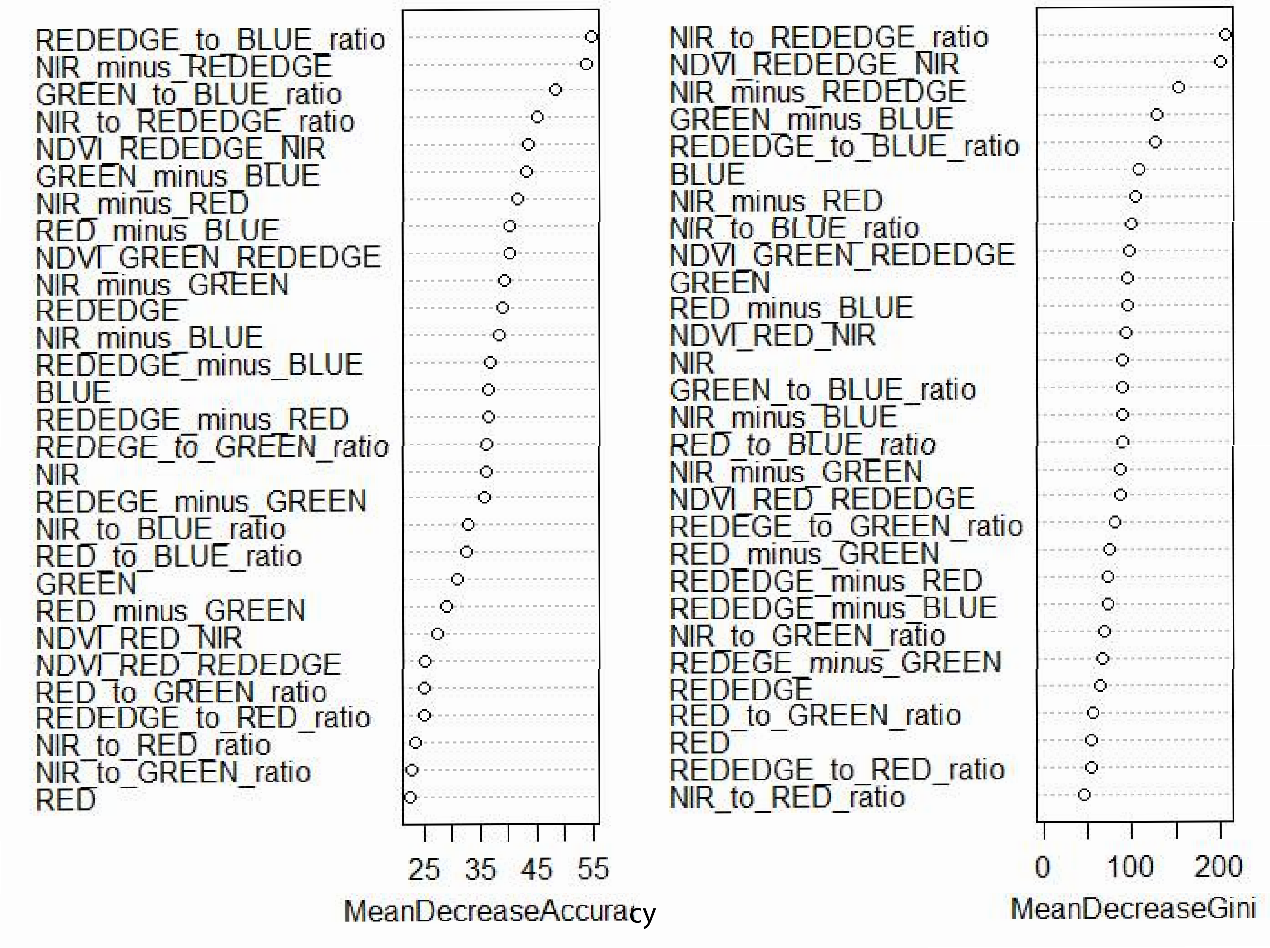

Mean Decrease in Accuracy (MDA) indicates the extent of decrease in accuracy in the Out-Of-Bag (OOB) samples when a variable is excluded from the predictive model. Variables with higher values of MDA are more important for classification. Mean Decrease Gini (MDG) defines the total decrease in Gini impurity (measure of datasets impurity) when a given variable is used for spliting at a node of all trees. Variables with higher MDA values are more important for classification (cf. Golino and Gomes 2014).

Reference

Golino, H. F., & Gomes, C. M. A. (2014). Visualizing Random Forest's Prediction Results. Psychology, 5(19), 2084.
