# Supplementary material for: Unveiling Undercover Cropland Inside Forests Using Landscape Variables: A Supplement to Remote Sensing Image Classification
Source: PLoS One. 2015 Jun 22;10(6):e0130079. doi: 10.1371/journal.pone.0130079 (PMC4476797; doi:10.1371/journal.pone.0130079)
Supplement: S2 Fig — (PDF) [file pone.0130079.s002.pdf]

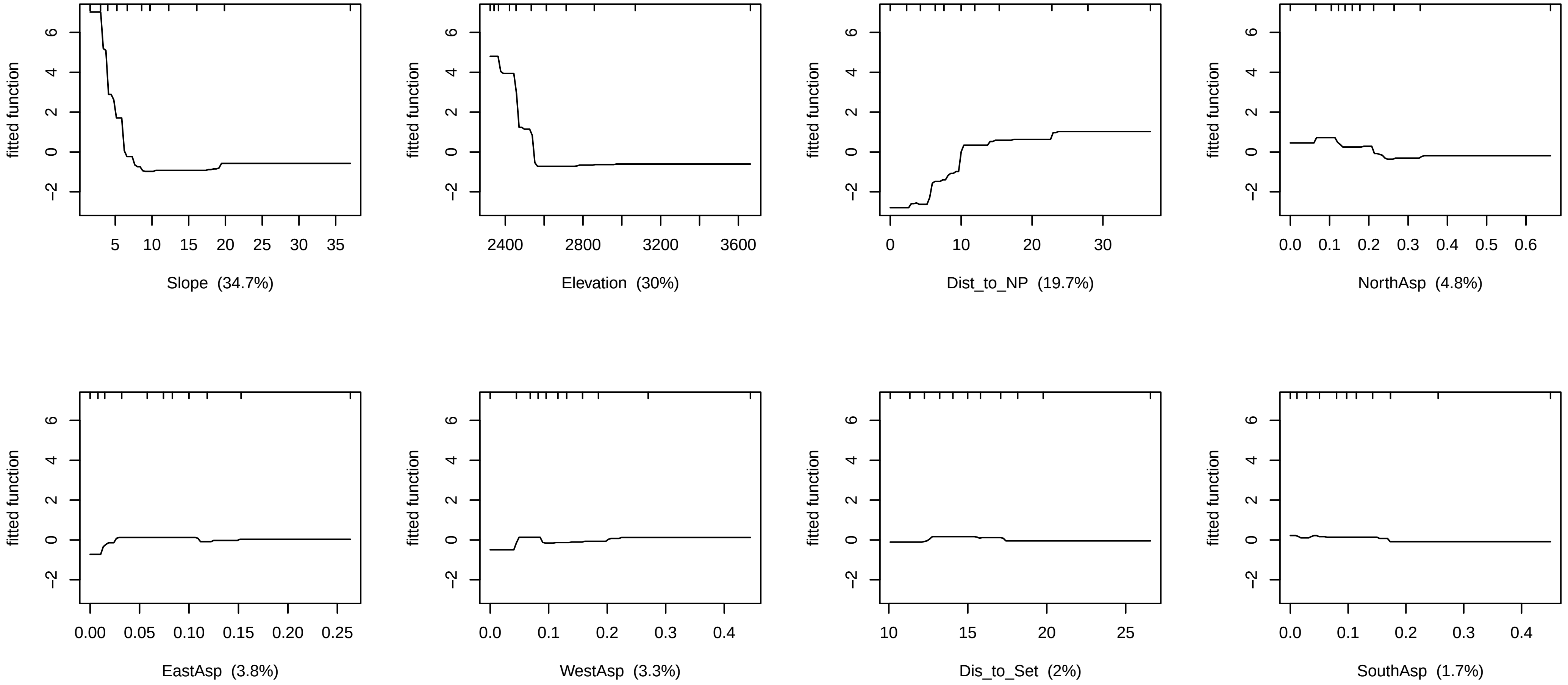

Variables with higher values are more influential. For instance, Slope and Elevation are more important than distance to settlements and South Aspect. Field estimated cropland area showed inverse relationship with Slopeand Elevation while it increases with increase in distance from the national park.

Key

* Dist_to_NP: Distance to National Parks; NorthAsp: North Aspect; EastAsp: East Aspect; WestAsp: West Aspect; Dis_to_Set: Distance to Settlements; SouthAsp: South Aspect
