# Supplementary material for: Unveiling Undercover Cropland Inside Forests Using Landscape Variables: A Supplement to Remote Sensing Image Classification
Source: PLoS One. 2015 Jun 22;10(6):e0130079. doi: 10.1371/journal.pone.0130079 (PMC4476797; doi:10.1371/journal.pone.0130079)
Supplement: S3 Fig — (PDF) [file pone.0130079.s003.pdf]

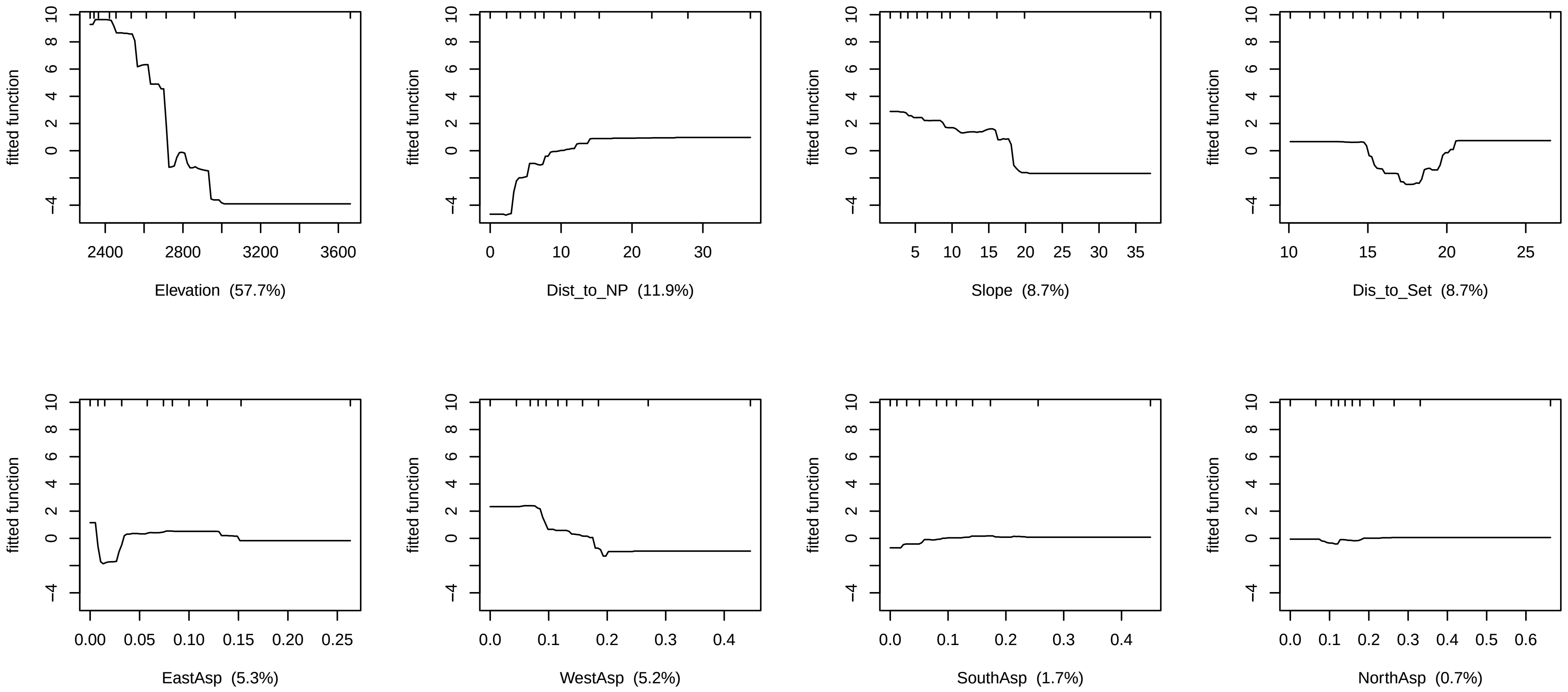

Variables with higher values are more influential. For instance, Elevation is more important than North Aspect. Cropland area calculated from RapidEye image classification showed inverse relationship with Elevation while itincreases with increase in distance from the national park.
