# Supplementary material for: Unveiling Undercover Cropland Inside Forests Using Landscape Variables: A Supplement to Remote Sensing Image Classification
Source: PLoS One. 2015 Jun 22;10(6):e0130079. doi: 10.1371/journal.pone.0130079 (PMC4476797; doi:10.1371/journal.pone.0130079)
Supplement: S4 Fig — Plots created using undercover cropland area calculated from field estimated percent cover (Fig a). Plots created using cropland area calculated from field estimated percent cover (Fig b). Plots created using cropland area calculated from RapidEye images (Fig c). (PDF) [file pone.0130079.s004.pdf]

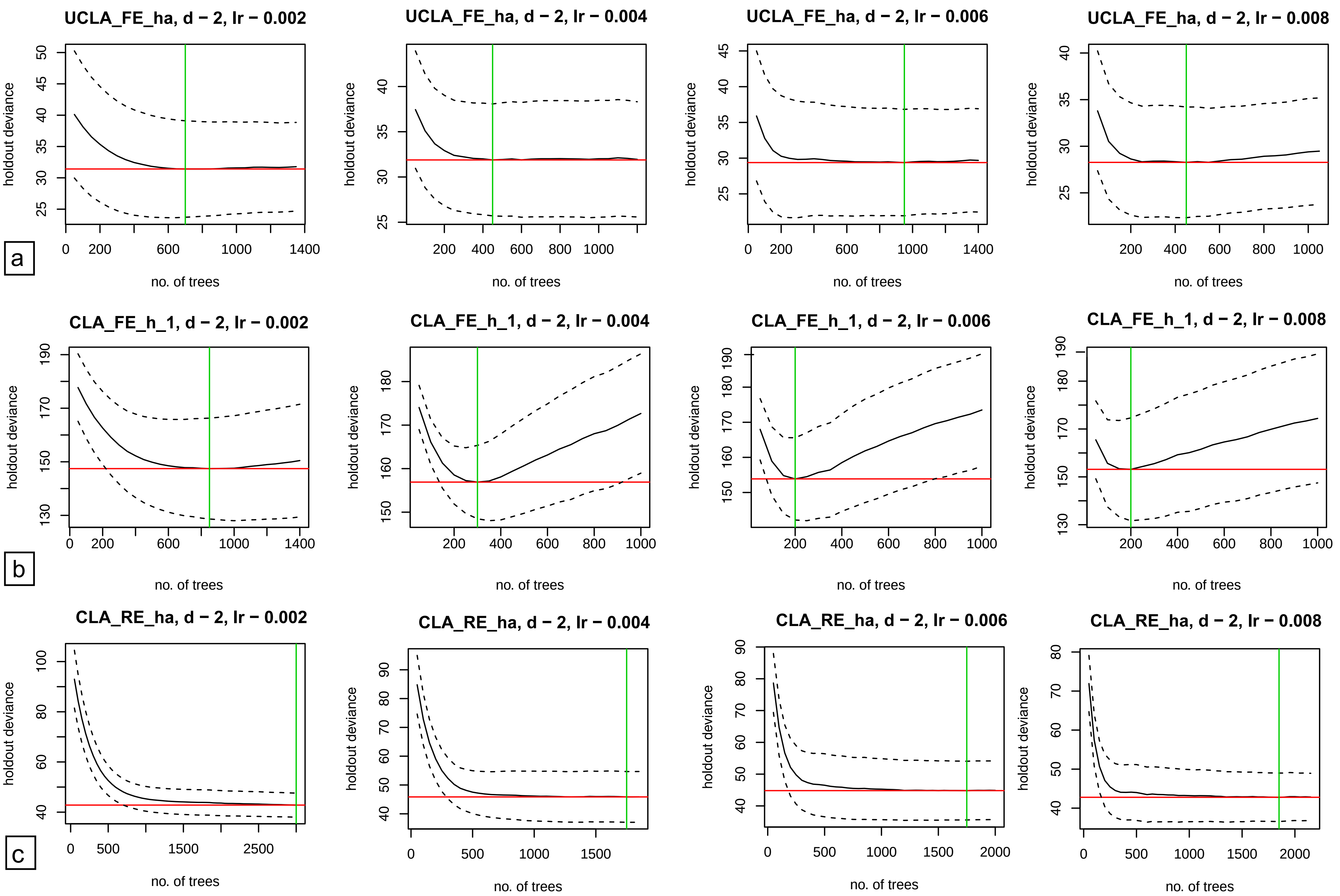

Key: UCLA_FE: Field Estimated Undercover Cropland; CLA_FE: Field Estimated Cropland Area; CLA_RE: Cropland Area calculated from RapidEye Images
