# Supplementary figures and images for: Unveiling Undercover Cropland Inside Forests Using Landscape Variables: A Supplement to Remote Sensing Image Classification
Source: PLoS One. 2015 Jun 22;10(6):e0130079. doi: 10.1371/journal.pone.0130079 (PMC4476797; doi:10.1371/journal.pone.0130079)

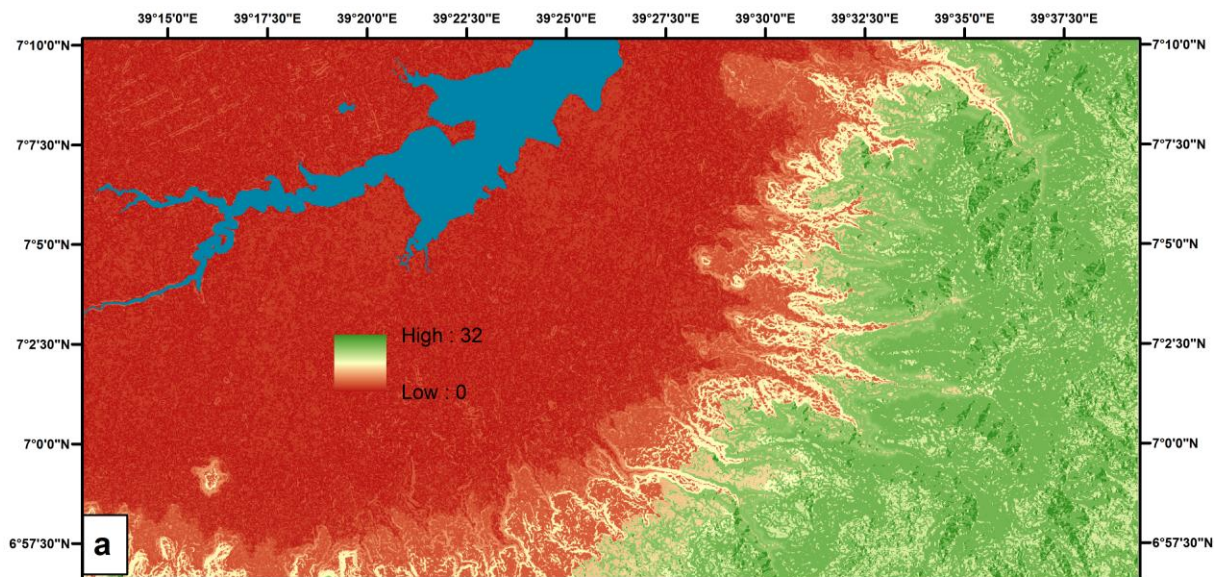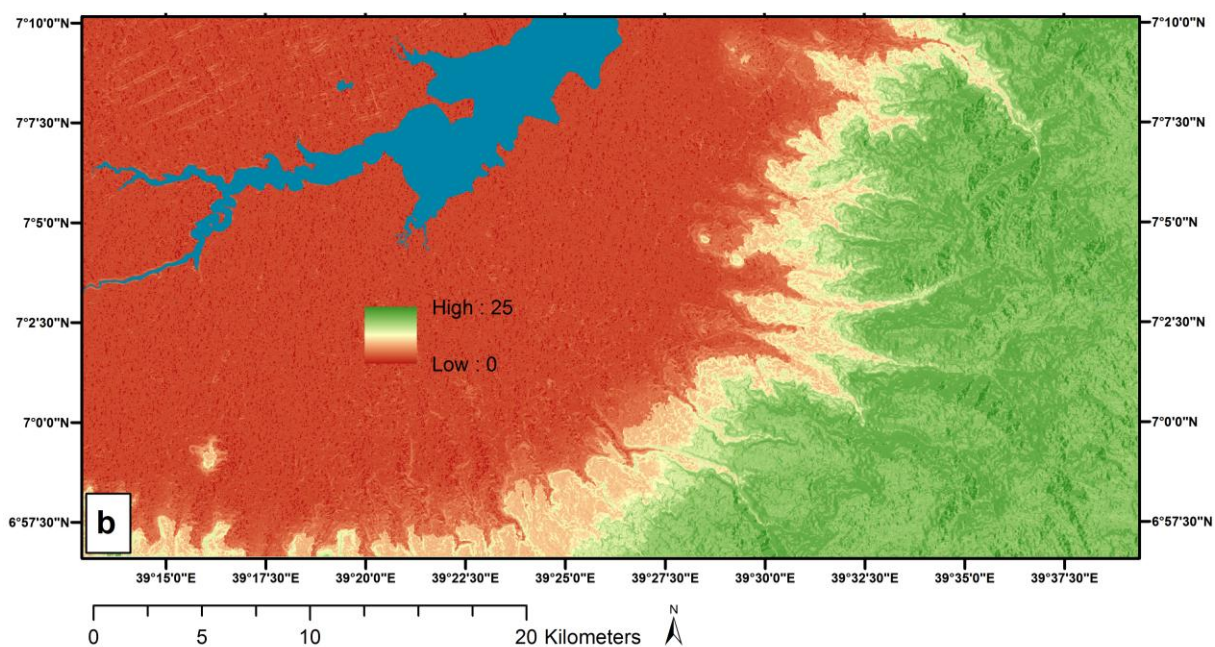

Supplement: S5 Fig — Prediction using only most influential factors slope, elevation and east aspect (Fig a). Prediction using all topographic factors slope, elevation, east aspect, west aspect, south aspect and north aspect (Fig b). (PDF) [file pone.0130079.s005.pdf]

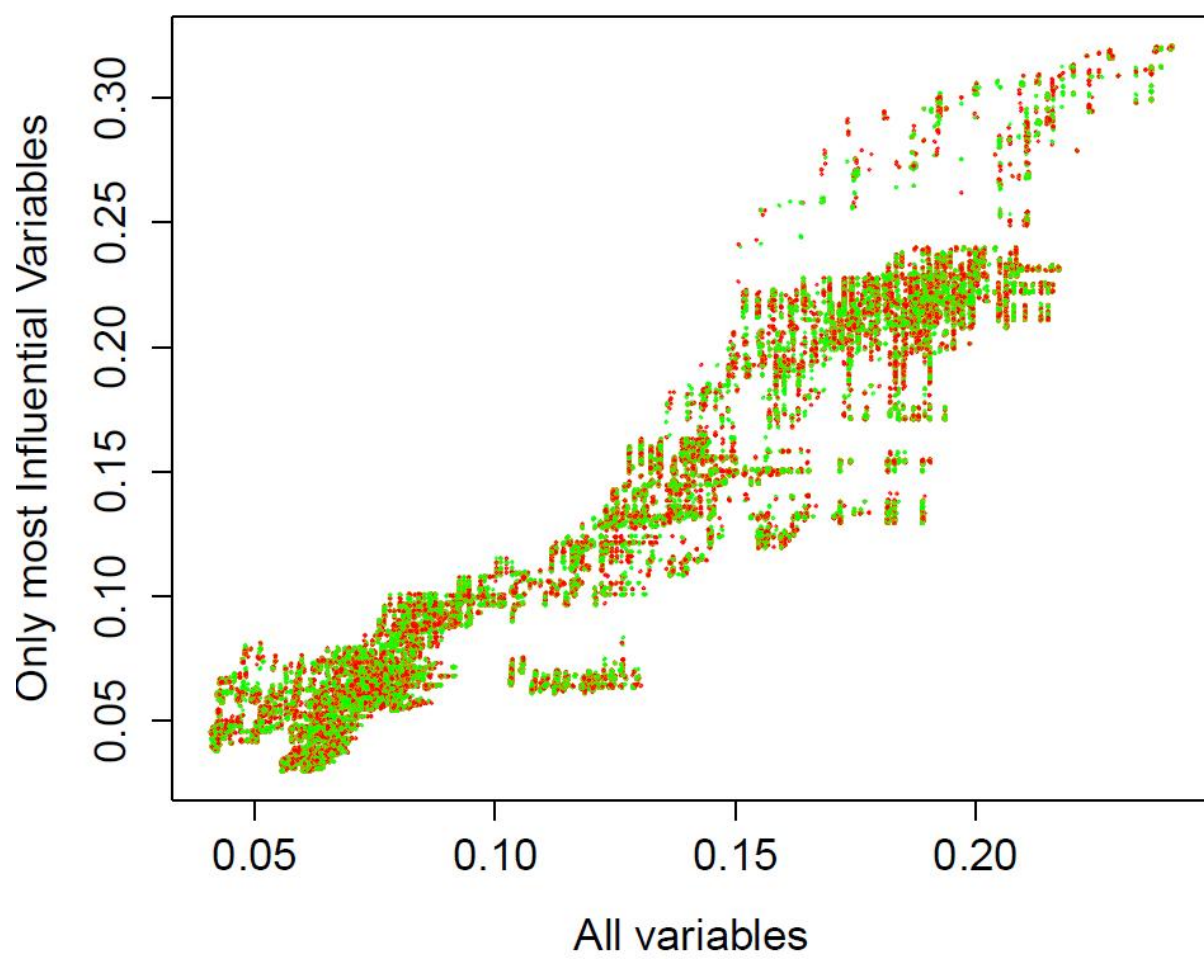

Supplement: S6 Fig — (PDF) [file pone.0130079.s006.pdf]

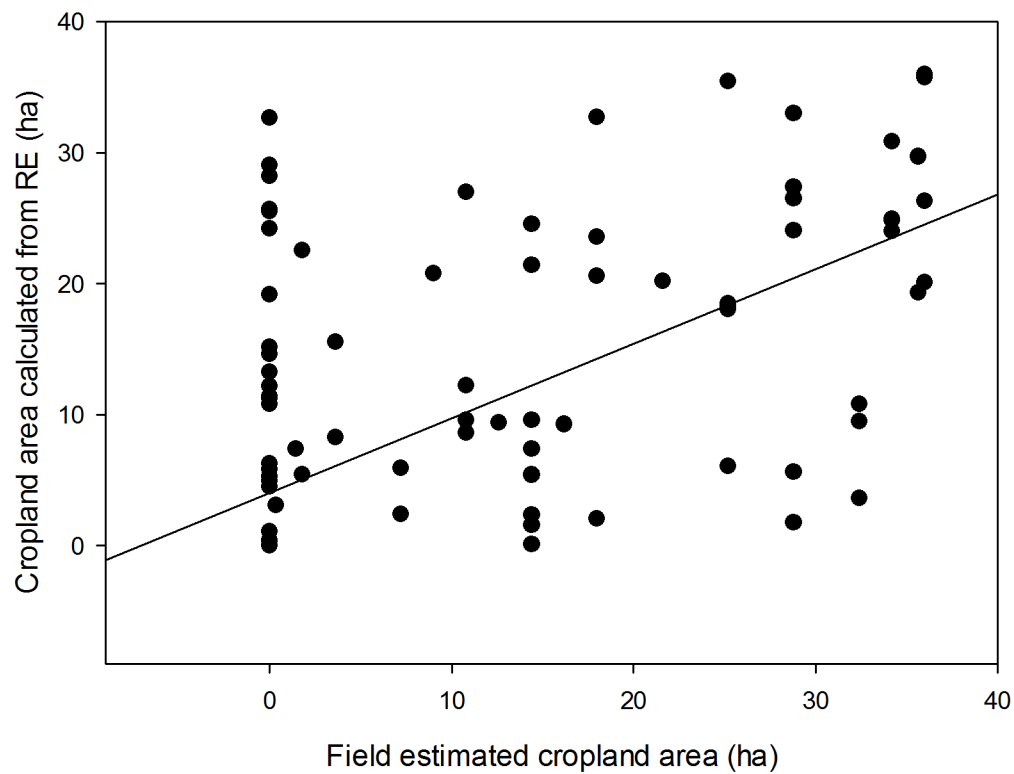

Supplement: S7 Fig — (PDF) [file pone.0130079.s007.pdf]

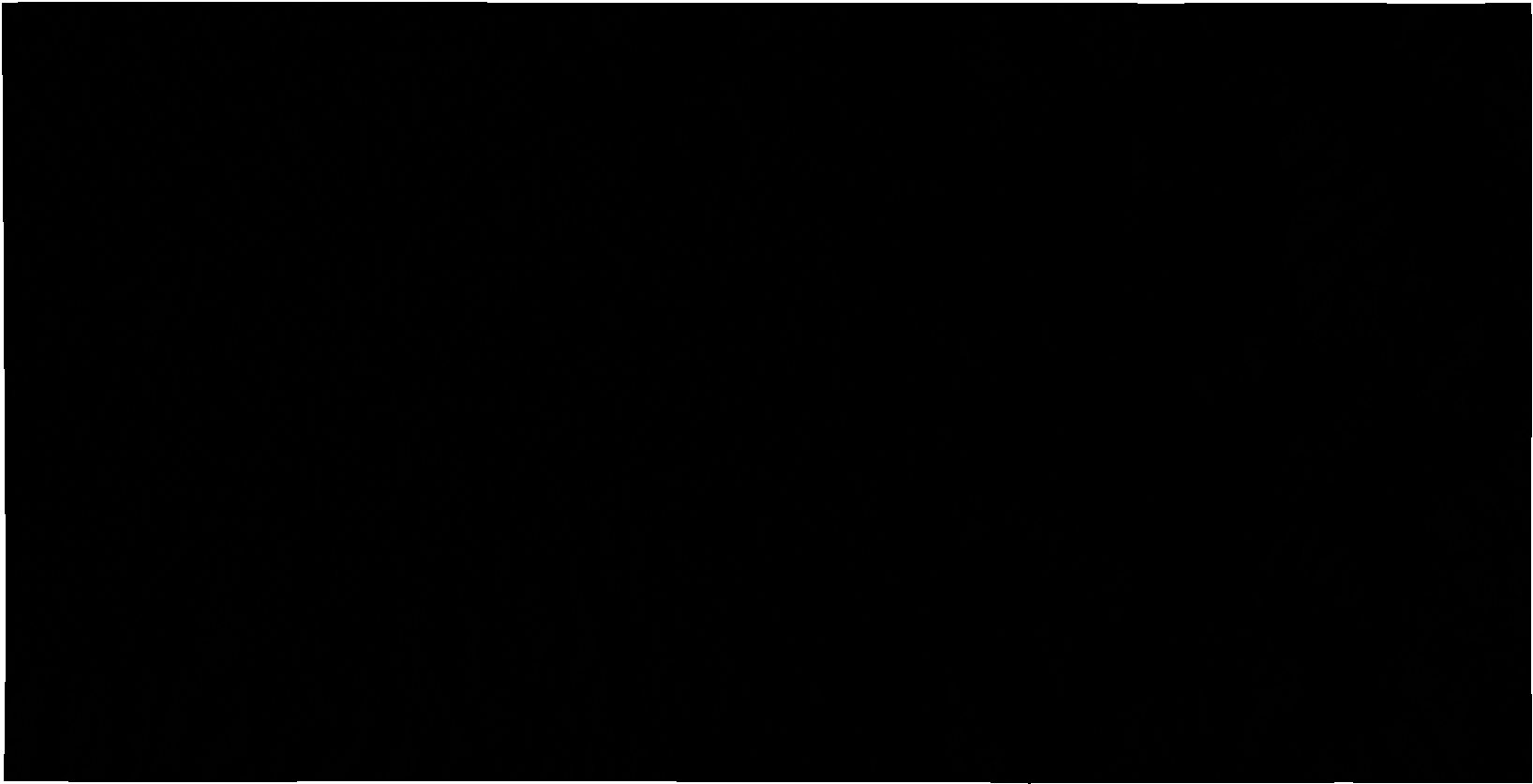

Supplement: S1 File — (ZIP) [file pone.0130079.s008.zip › Data/MainManuscript/Figure 3/Data/Raster Layers/eastaspect.tif]

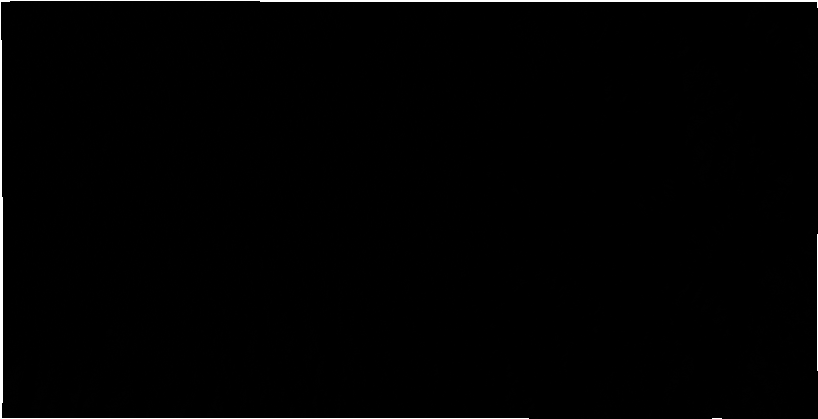

Supplement: S1 File — (ZIP) [file pone.0130079.s008.zip › Data/MainManuscript/Figure 3/Data/Raster Layers/eastaspect.tif.ovr]

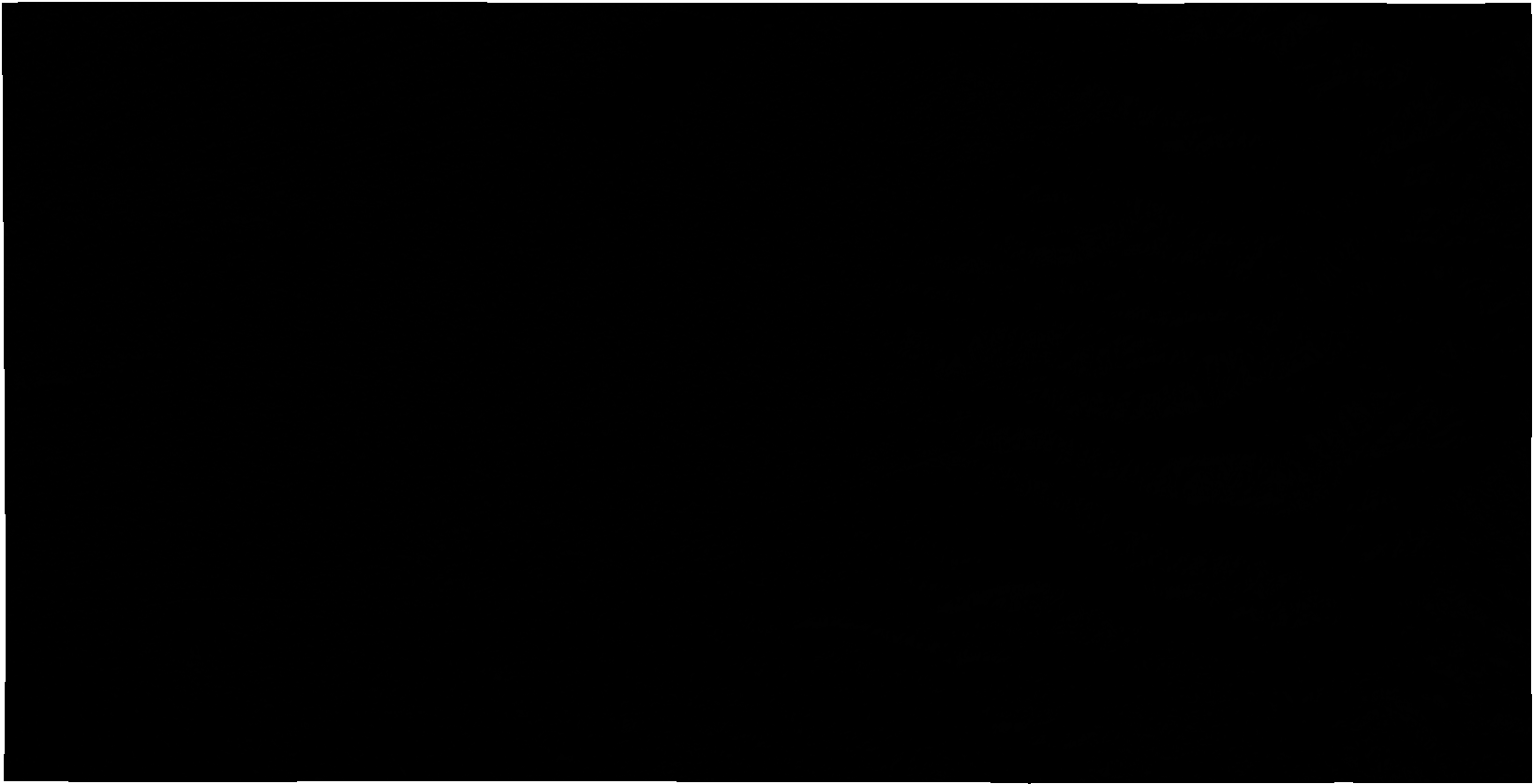

Supplement: S1 File — (ZIP) [file pone.0130079.s008.zip › Data/MainManuscript/Figure 3/Data/Raster Layers/southaspect.tif]

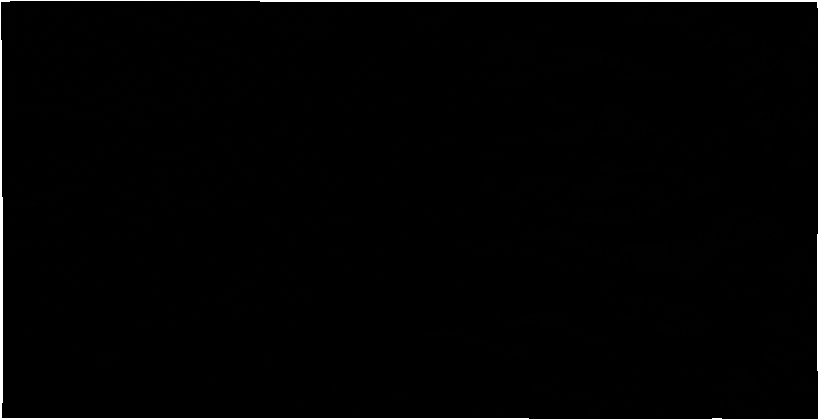

Supplement: S1 File — (ZIP) [file pone.0130079.s008.zip › Data/MainManuscript/Figure 3/Data/Raster Layers/southaspect.tif.ovr]

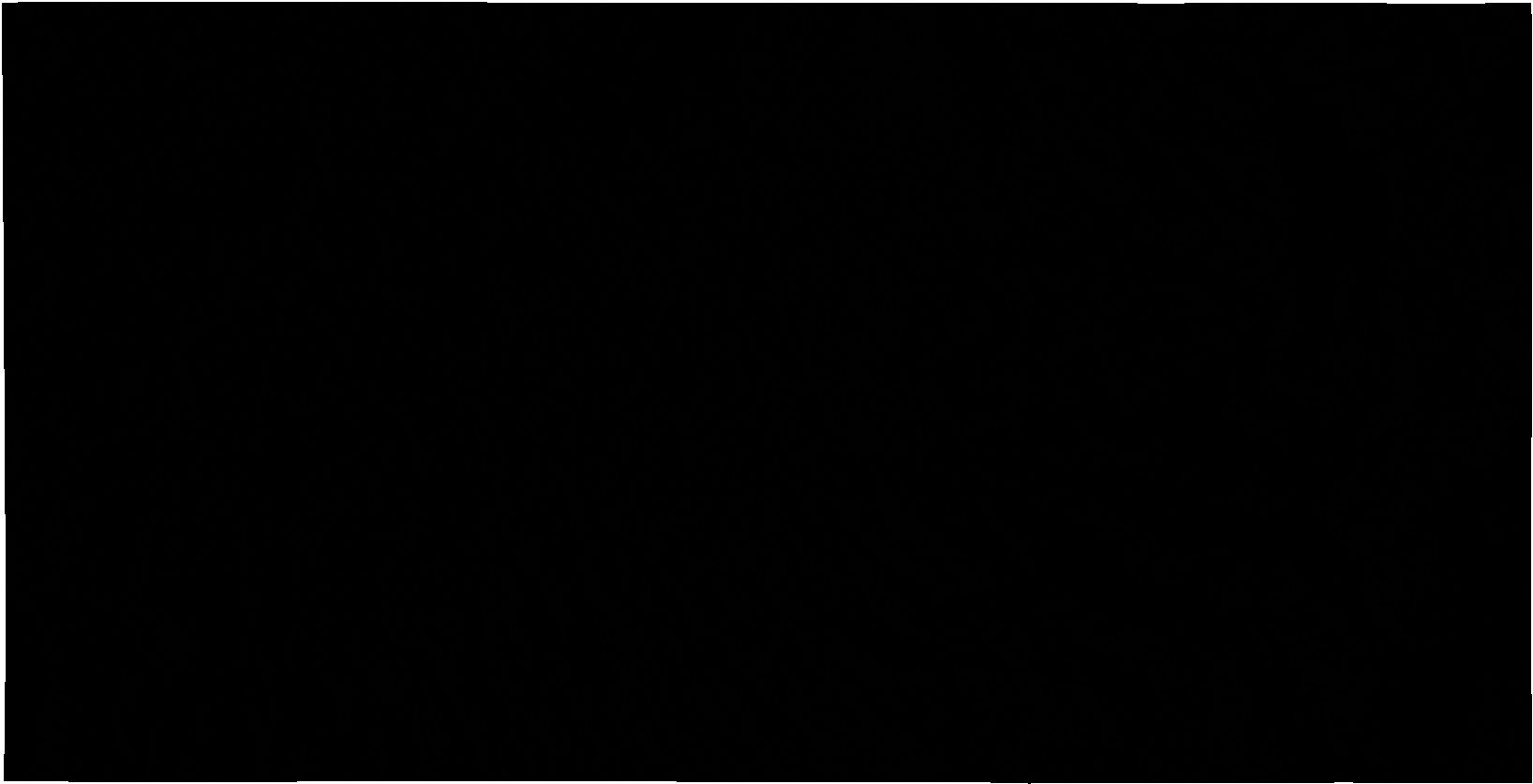

Supplement: S1 File — (ZIP) [file pone.0130079.s008.zip › Data/MainManuscript/Figure 3/Data/Raster Layers/westaspect.tif]

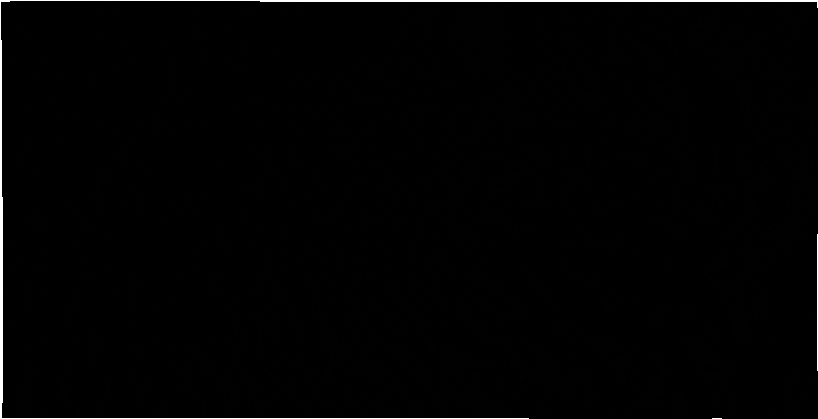

Supplement: S1 File — (ZIP) [file pone.0130079.s008.zip › Data/MainManuscript/Figure 3/Data/Raster Layers/westaspect.tif.ovr]

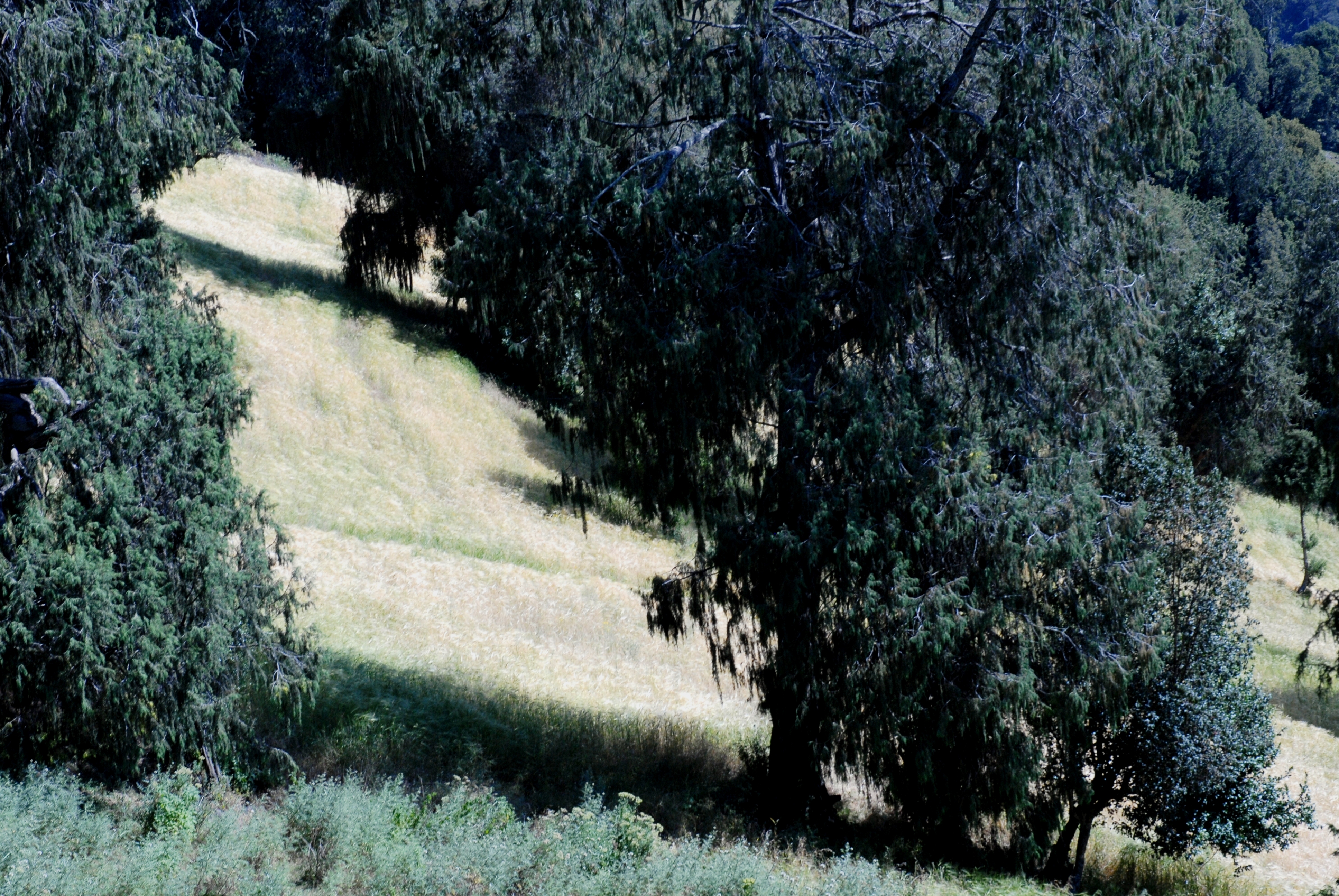

Supplement: S1 File — (ZIP) [file pone.0130079.s008.zip › Data/MainManuscript/Figure 4/Data/Photos/a.jpg]

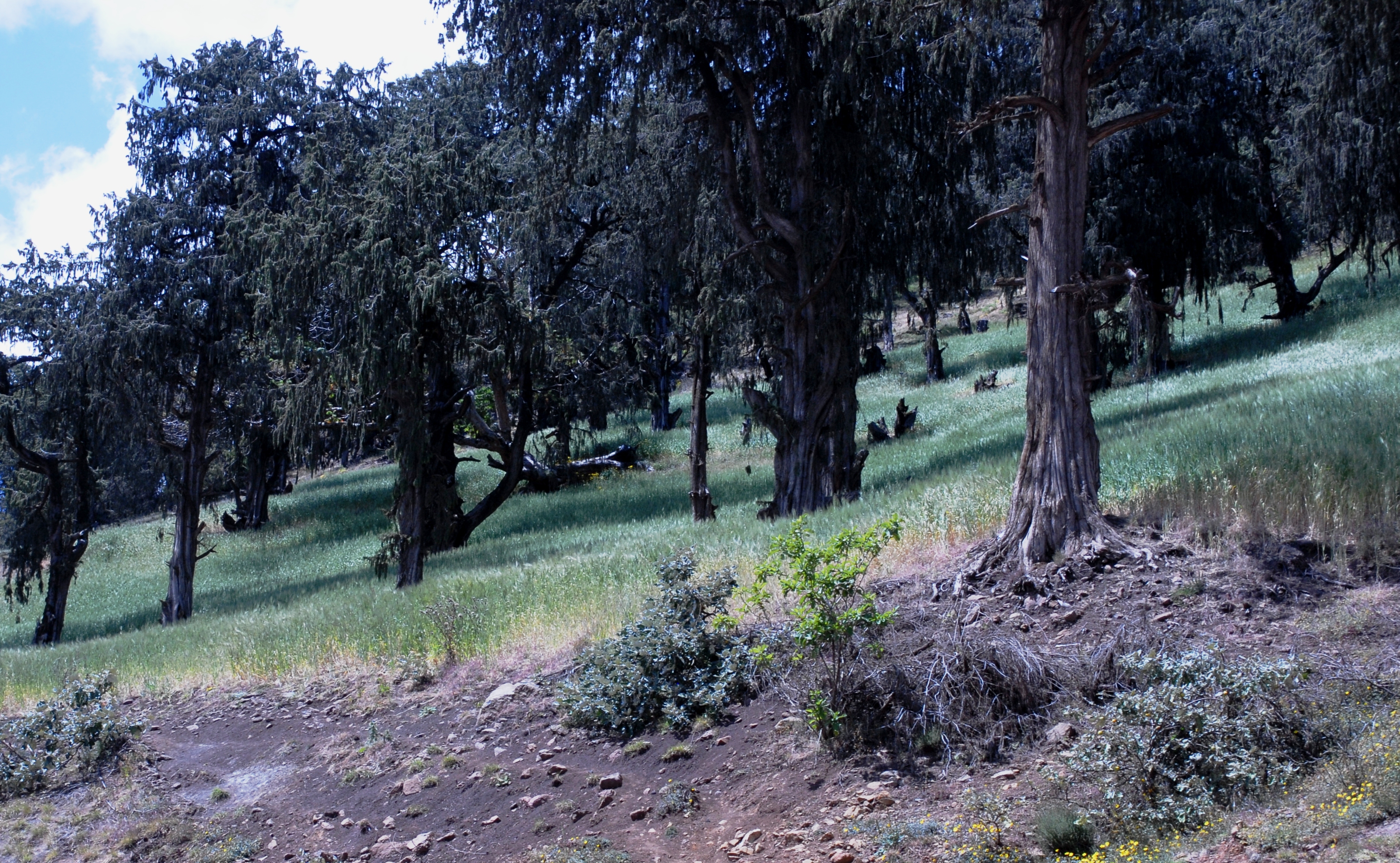

Supplement: S1 File — (ZIP) [file pone.0130079.s008.zip › Data/MainManuscript/Figure 4/Data/Photos/b.jpg]

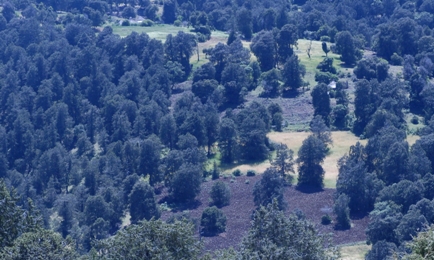

Supplement: S1 File — (ZIP) [file pone.0130079.s008.zip › Data/MainManuscript/Figure 4/Data/Photos/c.JPG]

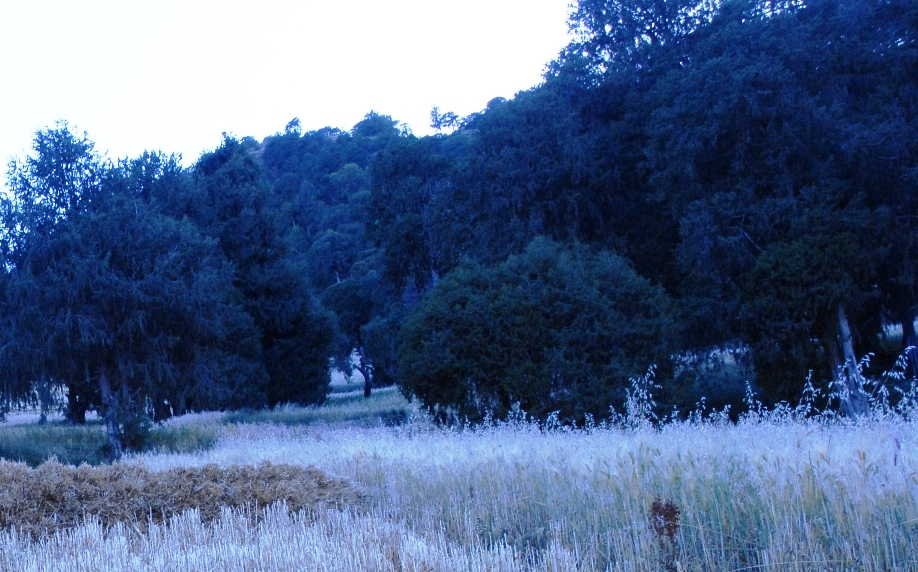

Supplement: S1 File — (ZIP) [file pone.0130079.s008.zip › Data/MainManuscript/Figure 4/Data/Photos/d.JPG]
